# Volcanic contribution to emergence of Central Panama in the Early Miocene

**DOI:** 10.1038/s41598-018-37790-2

**Published:** 2019-02-05

**Authors:** David M. Buchs, Derek Irving, Henry Coombs, Roberto Miranda, Jian Wang, Maurylis Coronado, Rodrigo Arrocha, Mauricio Lacerda, Creed Goff, Eladio Almengor, Enier Portugal, Pastora Franceschi, Eric Chichaco, Stewart D. Redwood

**Affiliations:** 1 School of Earth and Ocean Sciences, Cardiff University, Cardiff, UK; 2 Smithsonian Tropical Research Institute, Ancón, Panama City, Panama; 3 Ingineering Division, Panama Canal Authority, Corozal Oeste, Panama City, Panama; 4 Instituto de Geociencias, University of Panama, Colina Universidad de Panamá, Panama City, Panama; 5 Independant consulting geologist, Punta Pacífica, Panama City, Panama

## Abstract

Formation of the Panama Isthmus, that had global oceanographic and biotic effects in the Neogene, is generally associated with tectonic uplift during collision of the Panama volcanic arc with South America. However, new field, geochemical and geochronological data from the Culebra Cut of the Panama Canal suggest that volcanism also contributed to the Isthmus emergence in the Early Miocene. This volcanism is recorded in a newly-recognised Central Panama volcanic field that includes several phases of development. Early activity of this field along the Panama Canal was associated with proximal effusive to explosive felsic products during formation of subaerial stratovolcanoes and possible domes ca. 21 Ma. This was followed by a period of marine transgression ca. 21–18 Ma, with more distal volcanism documented by tuffs that deposited in marine to terrestrial environments. Finally, proximal mafic volcanism formed tephra cones in a monogenetic field ca. 18(-?) Ma. This was associated with phreatomagmatic processes in a coastal environment, with remarkable kilometre-wide subvolcanic peperitic intrusions. We propose based on these observations that formation of the Central Panama volcanic field was critical in shaping regional topography, and that this could have actively contributed to obstruction and closure of an interoceanic strait in Central Panama.

## Introduction

Formation of the Panama Isthmus is a significant geological event that is generally considered to have triggered global oceanic/climatic changes and inter-American migration of terrestrial organisms during the Great American Biotic Interchange (GABI) ca. 3 million years (Ma) ago^1–5^. Oceanographic constraints suggest that the Isthmus formed a complete inter-American land bridge at this time, and it has been postulated that this may have disrupted previous interoceanic current patterns and triggered the onset of glaciations in the northern hemisphere^6,7^. However, the geological record in Panama includes shallow-marine and terrestrial sedimentary deposits since at least the Late Eocene (ca. 45 Ma)^8–14^, with evidence for emergent (island) volcanoes since the Late Cretaceous (ca. 70 Ma)^15,16^. Discontinuous emergence and shallowing of the Panama volcanic arc in central and eastern Panama could have influenced palaeo-oceanographic patterns in the Caribbean and Atlantic since the Miocene, ca. 20–15 Ma (i.e., >10 m.y. before final emergence of the Isthmus)^17,18^. In addition, discontinuous emergence of the Isthmus could have facilitated migrations of terrestrial organisms between the Americas since the Miocene^2,19,20^. Alternatively, these migrations could have been primarily controlled by changing climatic and palaeo-environmental conditions in Central America and northern South America^21–23^. Overall, this suggests that there could be only a limited causal relationship between the emergence of Panama, palaeoceanographic changes and GABI. Fundamentally, indirect palaeoceanographic, biotic and tectonic proxies have commonly been used to date the formation of the Isthmus, but their interpretation in terms of palaeogeography remains strongly debated^5,18,24,25^. Therefore, it is critical to provide new field geological constraints to improve our understanding of the palaeogeographic evolution of the Isthmus and better evaluate its possible palaeoceanographic and biotic impacts.

In this study we present new field, geochemical and geochronological results from the Panama Canal that document distinct volcanic phases during the Early Miocene. We propose that the topographic development of Central Panama was influenced by these phases, and that volcanism likely played a significant role in obstructing and possibly closing an interoceanic strait in this area. Although formation of the Isthmus is generally interpreted to have formed due to tectonic collision of the Panama volcanic arc with South America, our results, combined with previous regional constraints, show that the emergence of Panama resulted instead from a complex interplay of constructional volcanism and a range of local to regional tectonic processes.

## Geological Setting of Central Panama

**Figure 1:**
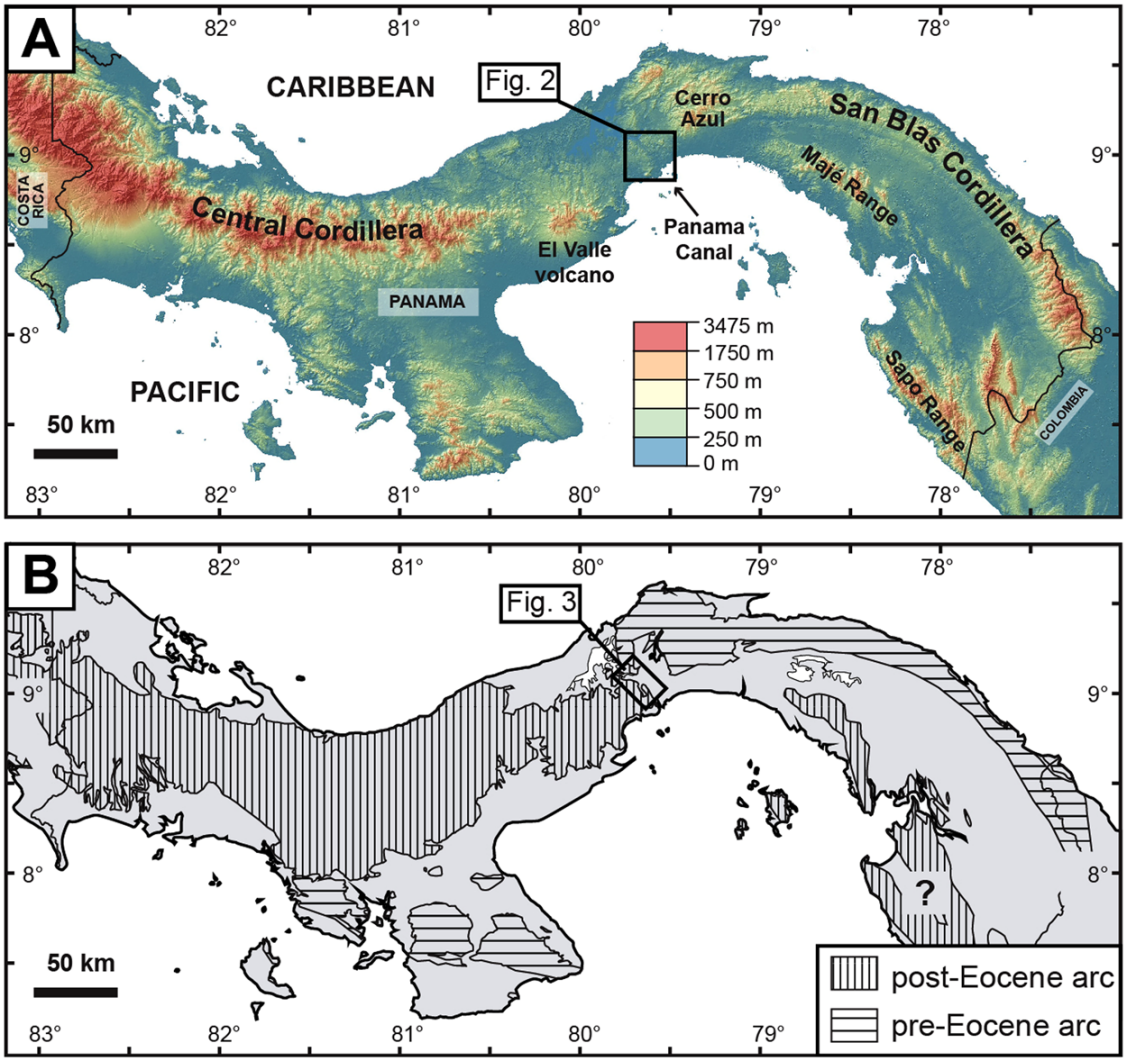
Main volcanic cordilleras of Panama outlined by (**A**) topography and (**B**) simplified geology. Digital topography model based on SRTM data retrieved online in 2017 with USGS Global Visualization Viewer tool (https://glovis.usgs.gov/).

**Figure 2:**
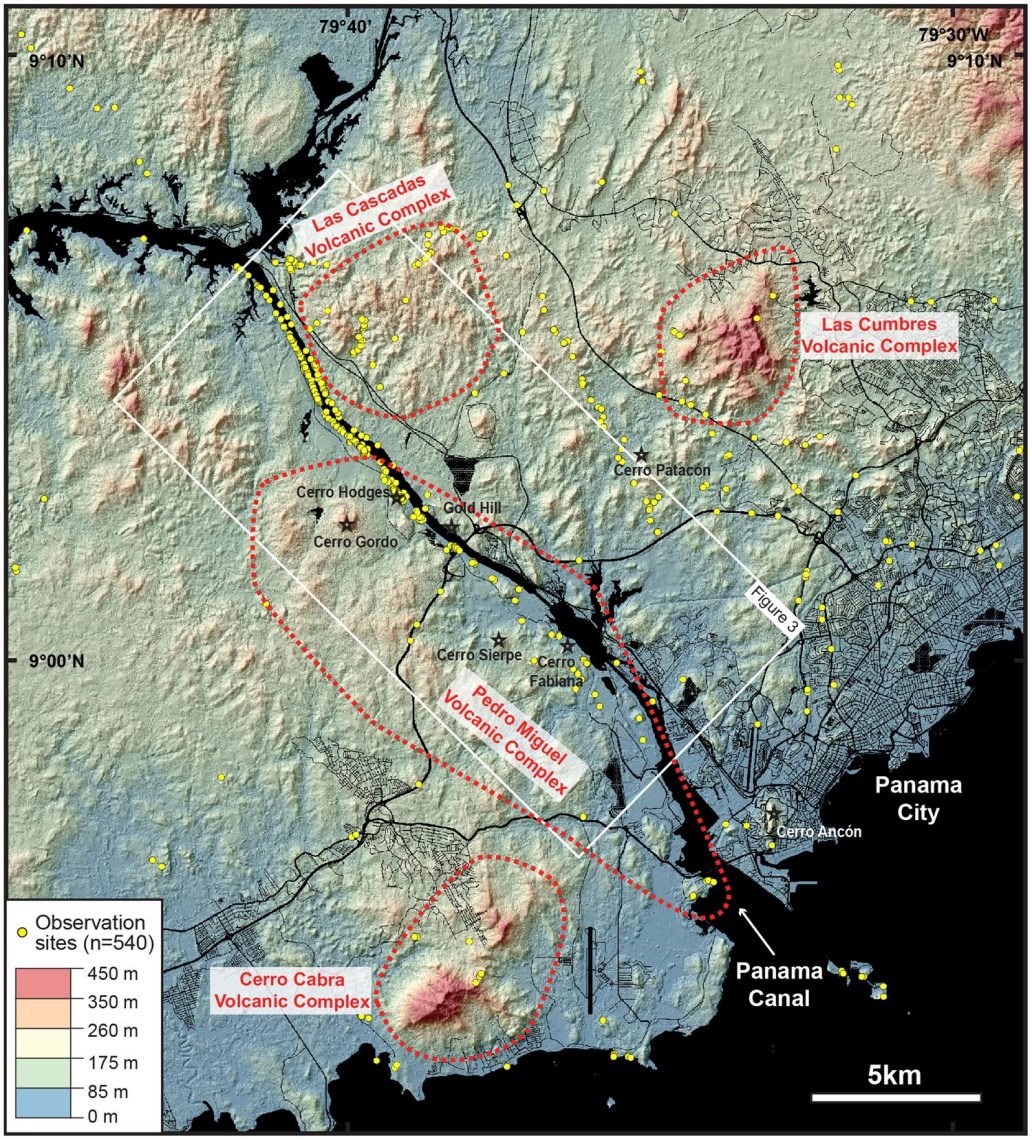
Topography of the Central Panama in the southern Panama Canal area and its main proposed volcanic complexes, with field coverage of this study (yellow dots). Digital topography model based on Lidar survey by the Panama Canal Authority. Roads and infrastructures are from Stamen Design (http://maps.stamen.com). Layers assembled with Open Source Geographic Information System QGIS (v. 2.18.19).

Central Panama, considered here as the area close to the Panama Canal between El Valle volcano and Cerro Azul mountains (Fig. 1A), is characterised by a unique geomorphology and geology in the Panama Isthmus. This area consists of lowlands punctuated by small (generally <300 m high) topographic features of unclear tectonic and/or volcanic origins (Fig. 2). It corresponds to a major topographic discontinuity between higher volcanic cordilleras that form the most prominent topography in the east and west (Fig. 1). Lineament analysis, geophysical constraints, palaeomagnetic data, and field observations show that the unique topography of Central Panama is at least in part controlled by a complex fault network associated with crustal-scale dismemberment of transisthmian volcanic cordilleras since the Late Eocene (ca. 38 Ma)^26–32^. However, it remains poorly constrained whether topographic highs in Central Panama represent erosional remnants of ancient volcanic landforms that could have obstructed/closed an interoceanic strait in this area.

**Figure 3:**
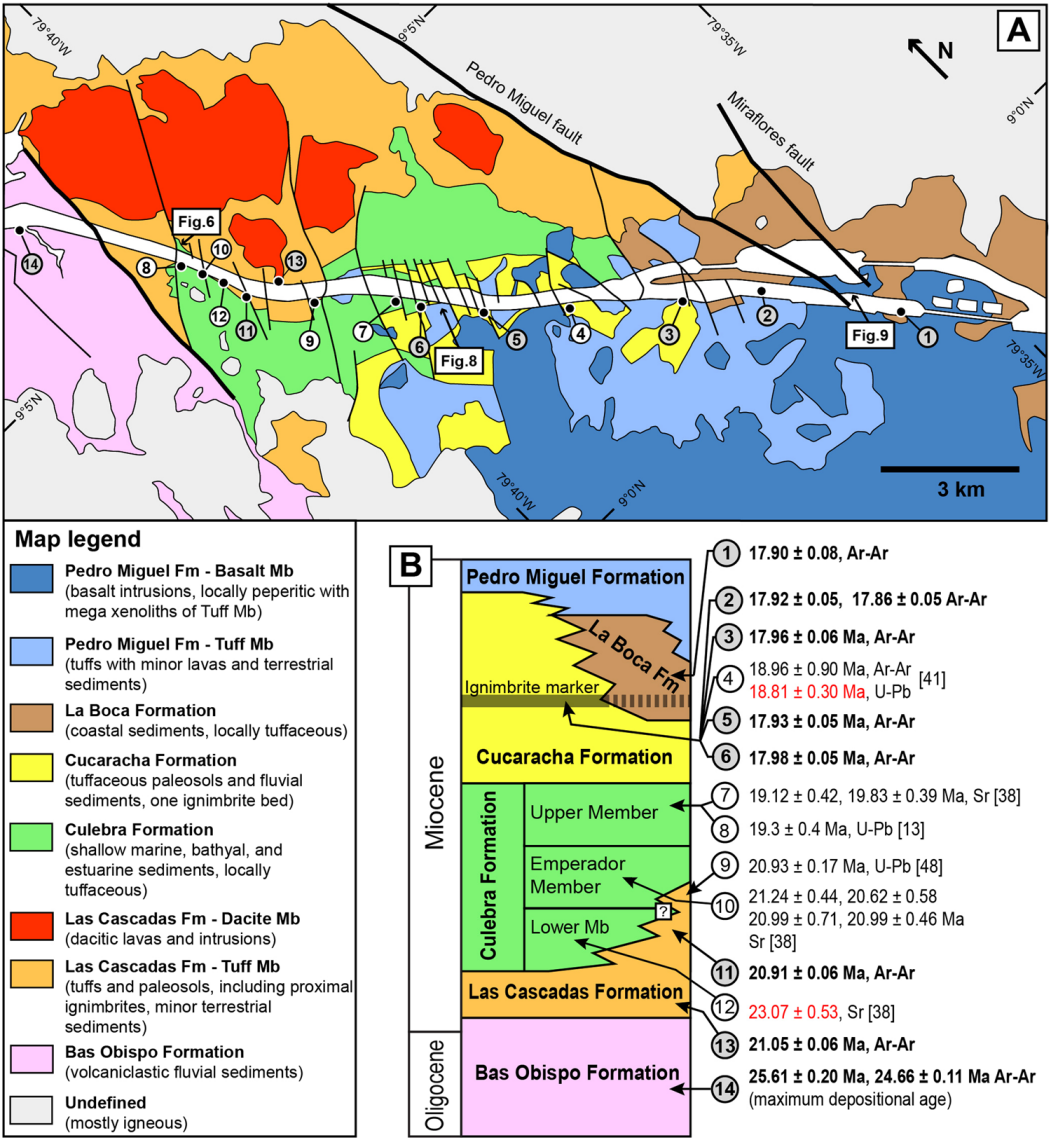
Geology of the southern part of the Panama Canal (Gaillard Cut and new Pacific locks area). (**A**) Revised geological map. (**B**) Revised chronostratigraphic chart with previous and new geochronological constraints (new data in dark circles and bold text).

Central Panama also corresponds to a major geological discontinuity along the Isthmus, with an extinct Late Cretaceous to Oligocene volcanic front in the east and two main volcanic fronts of Late Cretaceous to Eocene and Neogene ages in the west^33–36^ (Fig. 1B). In the simplest terms, the geology of Central Panama consists of a pre-Upper Eocene volcanic basement^34–36^ overlain by Upper Eocene to Pliocene marine to terrestrial sedimentary deposits interbedded with volcanic and volcaniclastic rocks^8,13,26,37–40^. Upper Eocene sedimentary deposits include shallow-marine limestones and tuffaceous mudstone-sandstone (Gatuncillo and Caimito Formations) that rest unconformably on top of an uplifted volcanic basement and mark a regional marine transgression^13,14,26,37^. Along the Gaillard (or “Culebra”) Cut of the Panama Canal (southern Central Panama, Figs 2 and 3), this Late Eocene transgression was followed by deposition of sediments and volcanic/volcaniclastic products in: (i) a terrestrial environment (Bas Obispo and Las Cascadas Formations, Oligocene to Early Miocene); (ii) shallow-marine to bathyal environments (Culebra Formation, Early Miocene); and (iii) a terrestrial environment (Cucaracha and Pedro Miguel Formations, Early Miocene)^13,17,26,37,38,40,41^. These sequences are followed by the deposition of younger marine sediments in northern Central Panama in the Late Miocene to Pleistocene (Alajuela, Gatun and Chagres Formations)^39,42,43^. Finally, a more recent marine regression possibly accompanied the first complete emergence of the Panama Isthmus^43^. Therefore, the geological record in Central Panama preserves at least 3 main cycles of marine transgression and regression since the Late Eocene. However, relationships between these cycles and volcanic and tectonic processes remain poorly constrained. Significantly, there is clear evidence for mafic to felsic volcanism in the Canal area between the Oligocene and Miocene (ca. 25 to 19 Ma)^13,35,40,44,45^. This volcanic activity is thought to be associated with particular magmatic processes during collision of the Panama volcanic arc with South America^44^. However, the volcanic evolution of this area and its possible links to the emergence of Central Panama remain to be characterised in detail.

Because of its low topography and local preservation of uplifted Eocene and younger marine deposits, Central Panama is generally considered to have hosted one of the last interoceanic straits before final emergence of the Isthmus^18,38,39,43,46^. This makes the geological record of the area particularly significant to characterise the possible role of volcanism in the emergence of the isthmus. This is addressed herein with new data from volcanic and volcaniclastic deposits exposed along the Gaillard Cut of the Panama Canal. This approach benefited from new exposures and cores associated with recent widening of the Panama Canal, with additional constraints from an extensive regional field survey (see Methods).

## Results

Our results reveal three main volcanic phases that span most of the geological record of the Gaillard Cut. We document below volcanic lithofacies and stratigraphic relationships associated with these phases. Main palaeo-environmental constraints are based on previous studies of associated sedimentary deposits. Our revised stratigraphic model (Fig. 3) builds upon original mapping by Canal geologists^26^, more recent local mapping in the Pedro Miguel Formation^40^, last stratigraphic revision of the Gaillard Cut^38^ and our new lithostratigraphic and geochronological constraints.

### Early volcanogenic sedimentation (Bas Obispo Formation)

**Figure 4:**
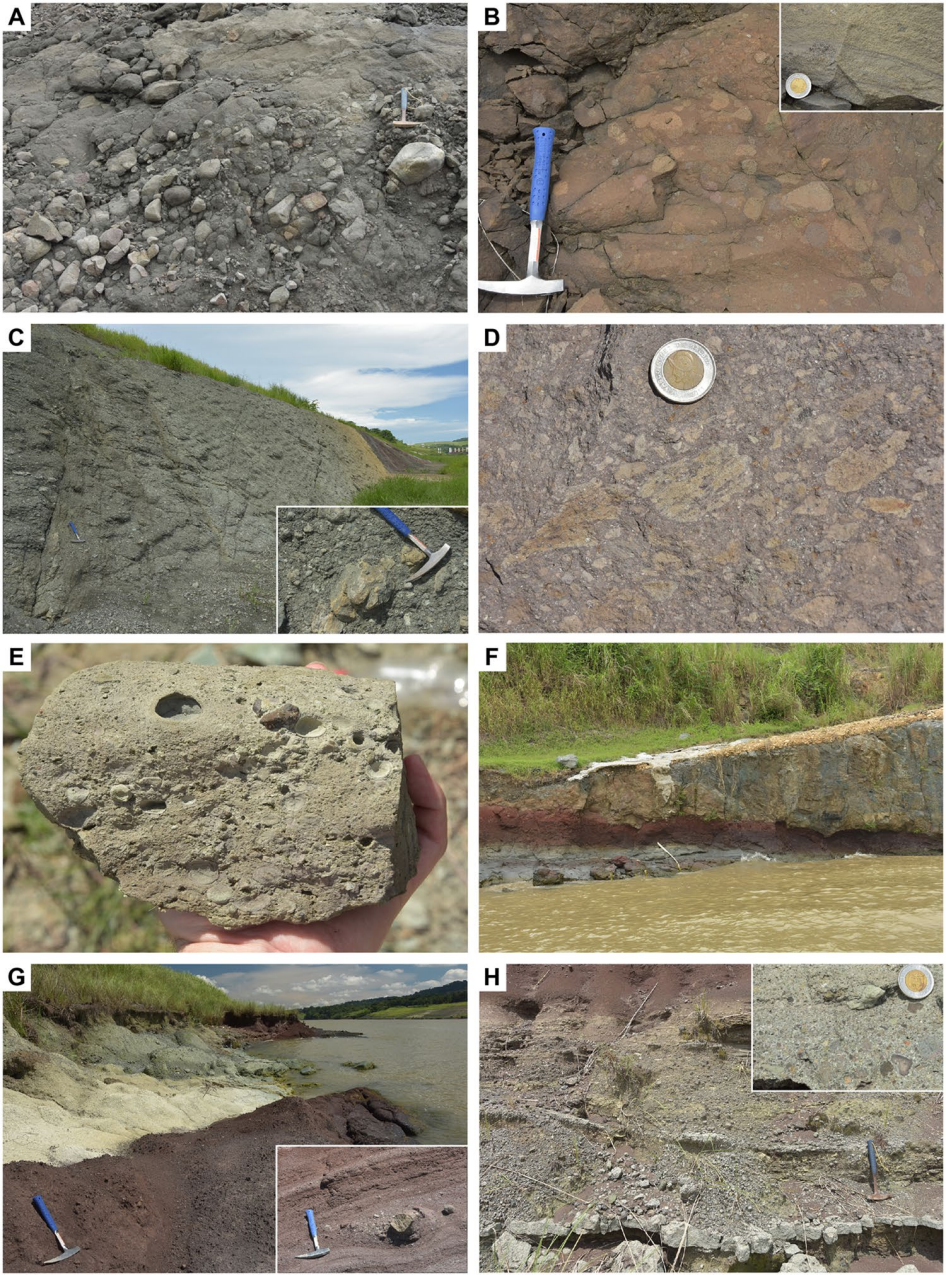
Selected volcanogenic lithologies of the Bas Obispo and Las Cascadas Formations (location of photos is given in Supplementary Dataset 1). (**A**) Volcanogenic fluvial conglomerate in the Bas Obispo Formation, with clast imbrication. (**B**) Layered volcanogenic pebbly sandstone in the Bas Obispo Formation. Inset shows mud drapes. (**C**) Lithic-rich pyroclastic density current deposit in the Las Cascadas Formation. Inset shows a large lithic of flow-banded dacite. (**D**) Pumice-rich pyroclastic density current deposit in the Las Cascadas Formation **(E)** Fallout coarse lapilli-tuff with accretionary lapilli in the Las Cascadas Formation. (**F**) Flow-banded dacite lava flow on top of a paleosol in the Las Cascadas Formation. Thickness of the flow is approximately 5 m. (**G**) Tuffaceous paleosols of the Las Cascadas Formation. Inset shows a lens of cross-bedded volcanogenic sedimentary breccia. (**H**) Cross-bedded volcanogenic sedimentary breccia and coarse sandstone in the Las Cascadas Formation, with clasts of amoeboid andesite/dacite in the inset.

The oldest sequences of the Gaillard Cut belong to the Bas Obispo Formation in the northern part of the Cut (Fig. 3). This unit precedes volcanic phases documented further south along the Canal. The Bas Obispo Formation was previously interpreted as an agglomerate^13,40,44^, but well-rounded imbricated pebbles and cobbles in new exposures unambiguously record the occurrence of bedload fluvial sediments throughout the unit (Fig. 4A). These deposits are interbedded with poorly layered, coarse pebbly sandstone that forms most of the unit, and are rarely associated with siltstones and mud drapes (Fig. 4B). These deposits are typical of debris to hyperconcentrated flows in a fluvial volcano-sedimentary environment^47^.

**Figure 5:**
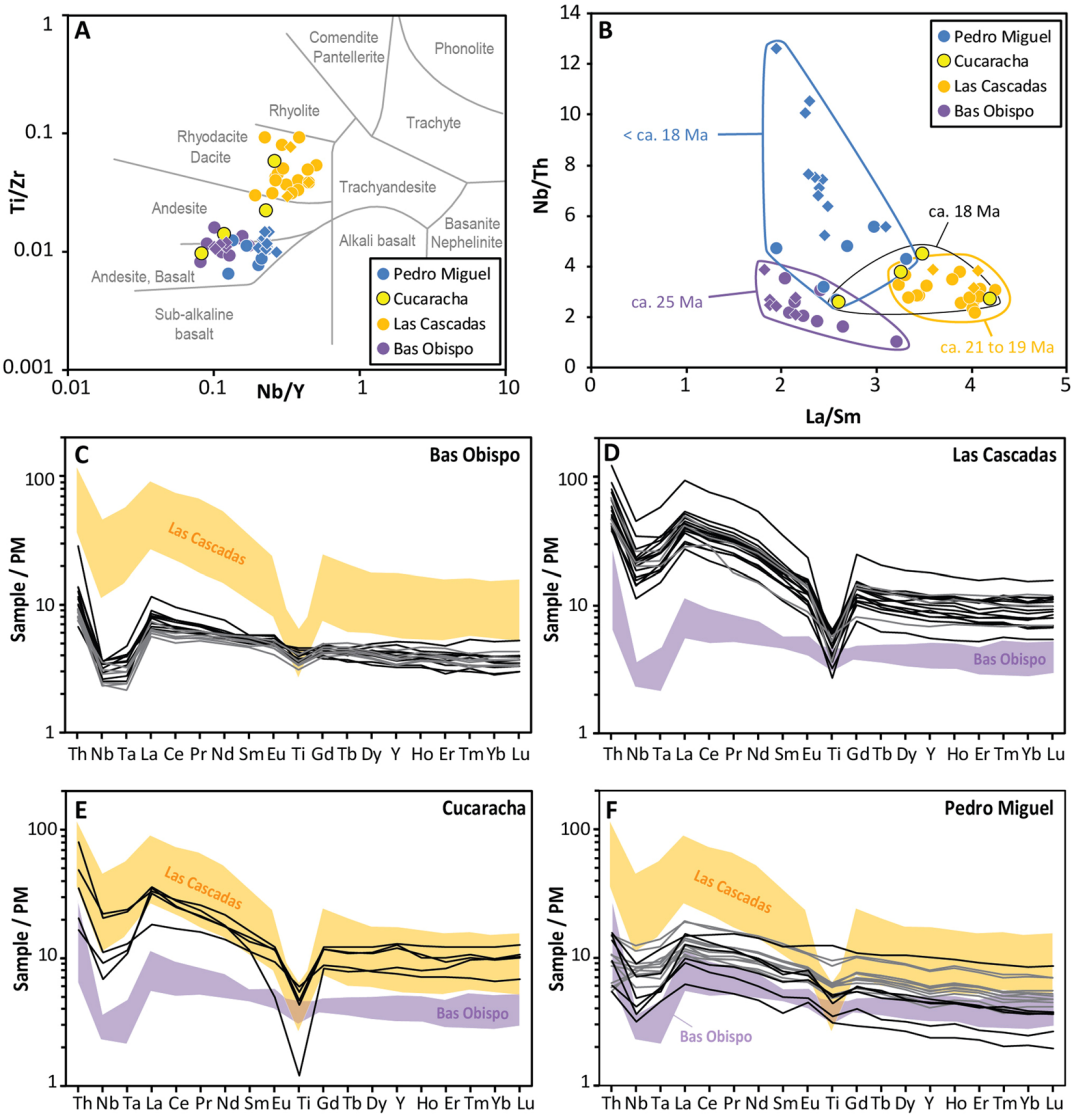
Geochemistry of volcanic and volcaniclastic rocks of the Gaillard Cut (location of analysed samples is given in Supplementary Dataset 1). (**A**) Nb/Y-Zr/Ti diagram from Winchester and Floyd^60^. Circles are for samples from this study and diamonds for samples from Farris *et al*.^40^. See Methods for details on data compilation. (**B**) La/Sm-Nb/Th diagram. Same symbols as in (A). (**C**–**F**) Primitive Mantle (PM)-normalised multielement diagrams. Black lines are for samples from this study and grey lines for samples from Farris *et al*.^40^. Primitive mantle values are from McDonough and Sun^65^.

The Bas Obispo sandstone is an immature lithic arenite with angular fragments of andesite, feldspar, clinopyroxene and opaque minerals (no fossil or amphibole in our samples). The clasts in the sandy matrix are composed of porphyritic andesite with multiply zoned plagioclase and clinopyroxene (no amphibole in our samples). Lithification of the Bas Obsipo Formation is highly variable (hard to crumbly) due to heterogenous cementation by authigenic clay (no volcanic welding). The andesite clasts have a very consistent geochemical signature which is distinct from other units of the Gaillard Cut (Fig. 5). These observations indicate that this sedimentary unit was formed by proximal reworking from a volcanic sequence without younger equivalent along the Gaillard Cut.

The age of emplacement of the Bas Obispo Formation is constrained by: (i) 25.61 ± 0.20 and 24.66 ± 0.11 Ma Ar-Ar ages of two clasts of andesites; and (ii) a crosscutting felsic dyke (sample D16-013) of the Las Cascadas Formation of ca. 21 Ma age (see below). Based on these constraints, the Bas Obispo Formation was deposited in the latest Oligocene-earliest Miocene (24.6–21.1 Ma). This formation could grade laterally to the terrestrial to shallow-marine deposits of the Bohio Formation and lower to middle members of the Caimito Formation in the Lake Gatun area^42^.

### Proximal felsic volcanism (Las Cascadas Formation)

The first *in situ* volcanic phase of the Gaillard Cut is documented by the Las Cascadas Formation that overlies the Bas Obispo unit in the central part of the Cut (Fig. 3). This unit was previously described as a sequence of subaerial felsic tuffs of poorly defined volcanic origin, andesite lavas, obsidian, agglomerates, volcano-sedimentary deposits and paleosols^13,40,48^. New and previous geochemical data show that volcaniclastic deposits and lavas have a distinctive, similar dacitic composition that attests for a common magmatic origin of the studied sequences (Fig. 5).

**Figure 6:**
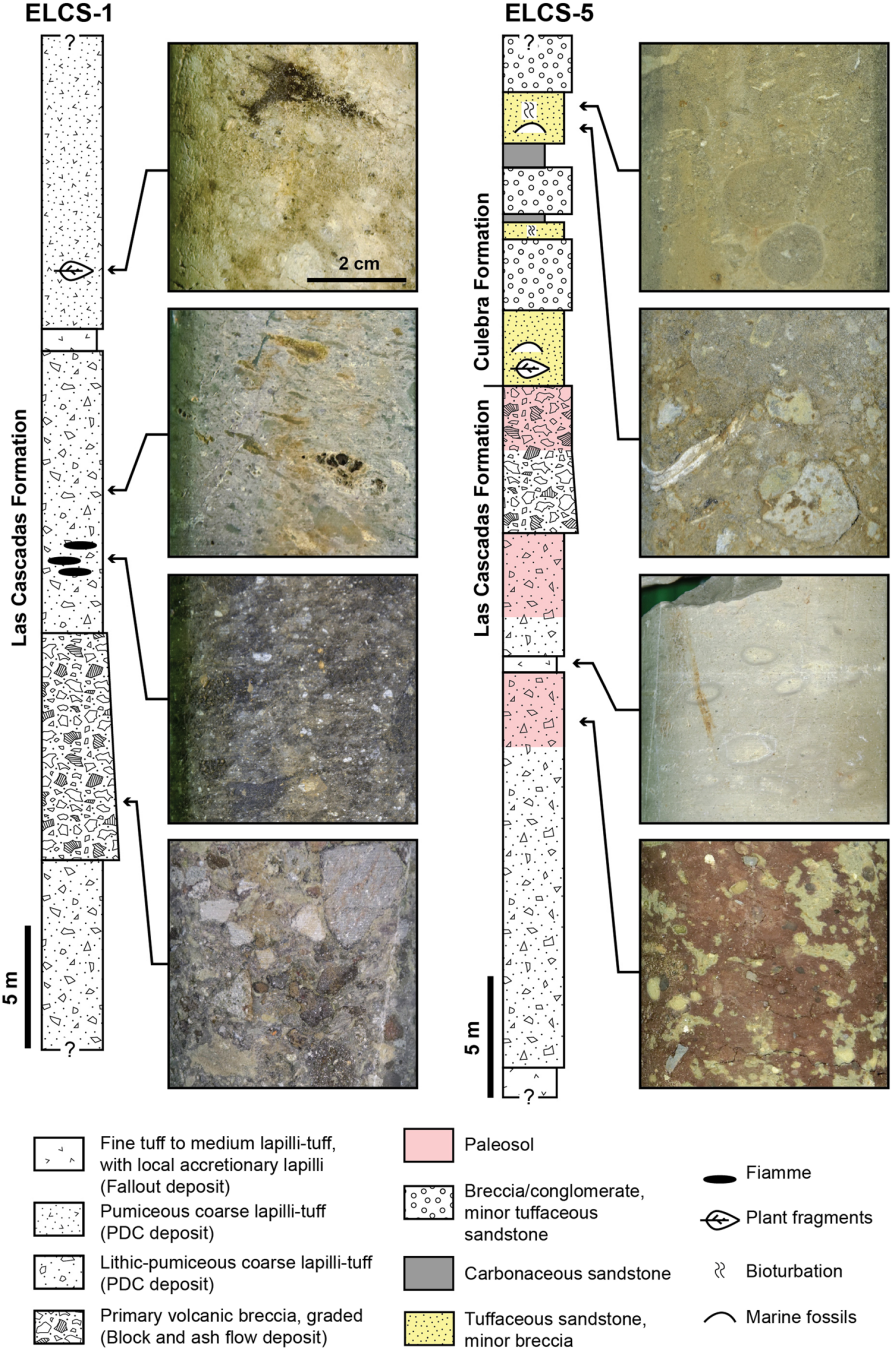
Typical lithostratigraphic assemblages from the Las Cascadas Formation. (**A**) Log of core ELCS-1 showing a typical ignimbrite succession. (**B**) Log of core ELCS-5 showing the transition between the Las Cascadas and Culebra Formations (approximative location of cores is given in Fig. 3).

The Las Cascadas Formation is characterised by an abundance of welded pyroclastic density currents (PDC) deposits, or ignimbrites, that are generally thick (locally > 10 m). These ignimbrites typically include high abundance of large lithics (e.g., flow-banded dacite) and/or flattened pumices (>40% volume) (Figs 4C,D and 6), with only minor (<10% volume) pyroxene and plagioclase phenocrysts embedded in an eutaxitic matrix. Rarely, the ignimbrites have a base rich in fiamme (Fig. 6), which was probably misinterpreted for obsidian lavas in previous studies. Ignimbrites locally include carbonized plant fragments (Fig. 6). Some graded lithic-rich tuffs are interpreted as block-and-ash flow deposits (e.g., second bed from bottom in Core ELCS-1, Fig. 6). Significantly, preceding observations indicate that most of the ignimbrites emplaced through proximal, concentrated PDCs^49^.

The Las Cascadas ignimbrites are commonly interbedded with fallout fine tuff to coarse lapilli-tuff, with locally large cm-sized accretionary lapilli and intermediate-felsic lithics (Figs 4E and 6). These deposits are associated with red to white tuffaceous paleosols that often preserve an original pumiceous fabric or are mottled (Figs 4F–H and 6). The paleosols are locally interbedded with lenses of cross-stratified sandstone to breccia, with abundant clasts of dacites and occasionally amoeboid/juvenile andesite (?) clasts (Fig. 4G,H). Agglomerates (used here in the more common sense of proximal vent deposits/spatter) were not observed, but we found rare possible occurrences of channelized lahar deposits. All of the preceding tuffaceous deposits are grouped here in the newly-defined Tuff Member of the Las Cascadas Formation (Fig. 3). This unit corresponds to the original Las Cascadas Formation on the regional geological map^26^ and could extend laterally to the upper (tuffaceous) member of the Caimito Formation in the Lake Gatun area^42^.

Other volcanic deposits in the Las Cascadas Formation consist of flow-banded greenish dacite lavas that are restricted to the NE side of the central Gaillard Cut (Fig. 4F), and rare brecciated dacite dykes in fault zones. These rocks are interbedded with, or crosscut, tuffs similar to those described above. The dacite lavas can be followed to the NE of the Canal in river exposures, where they are associated with dacitic-rhyolitic intrusions and correlate to a topographic high (Figs 2 and 3). These dacite-rich sequences are part of a newly-defined Dacite Member of the Las Cascadas Formation (Fig. 3), which replaces some of the undifferentiated felsic igneous units on the original geological map^26^.

New Ar-Ar ages of a tuff (20.91 ± 0.06 Ma) and lava flow (21.05 ± 0.06 Ma) are consistent with a 20.93 ± 0.17 Ma U-Pb zircon age of a tuff in the upper part of Las Cascadas Formation^48^. This indicates that this unit was quickly deposited in the Early Miocene (ca. 21 Ma). Although detailed stratigraphic relationships along the Canal are obscured by faults, new cores (e.g., ELCS-1 and ELCS-5, Fig. 6) suggest that there is significant lateral variability within the Las Cascadas Formation. Overall, geochemical, lithofacies, geomorphological, and geochronological constraints from the Las Cascadas Formation support existence of an Early Miocene volcanic complex in the NE of the Gaillard Cut, introduced here as the “Las Cascadas Volcanic Complex” (Fig. 2). Volcanic and volcaniclastic observations indicate that this complex is composed of subaerial stratovolcano(es) that were associated with explosive volcanism most likely during dome collapses to Plinian eruptions.

### Distal subaerial volcanism (Culebra, Cucaracha and La Boca Formations)

**Figure 7:**
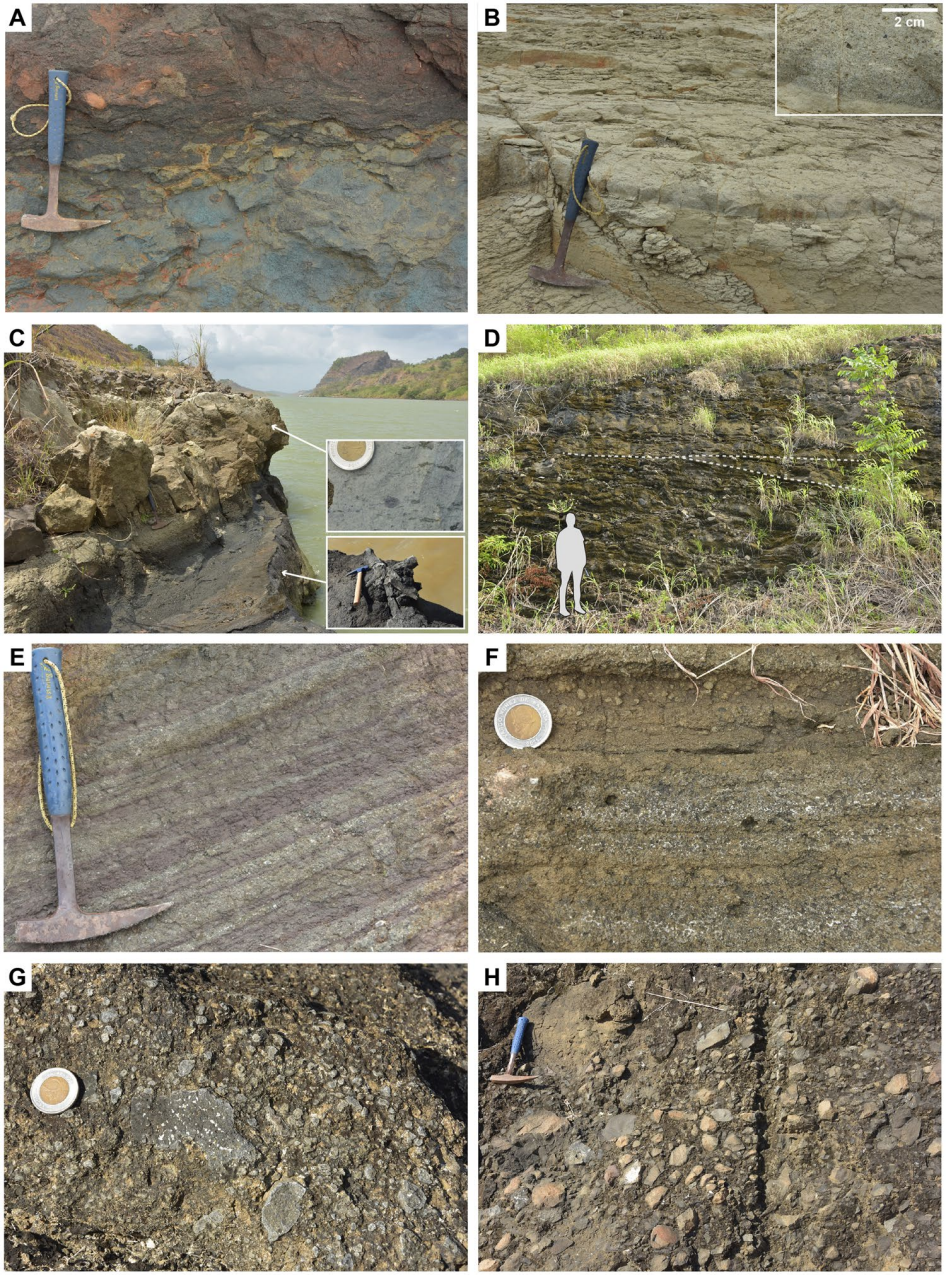
Selected volcanogenic lithologies of the Culebra, Cucaracha and Pedro Miguel Formations (location of photos is given in Supplementary Dataset 1). (**A**) Tuffaceous sediment in lagoonal, Lower Member of the Culebra Formation. (**B**) Tuffaceous turbidites in a bathyal, lower Upper Member of the Culebra Formation. (**C)** Ignimbrite of the Cucaracha Formation. Insets show the eutaxitic texture of the ignimbrite and charcoaled tree stump below the ignimbrite. (**D**) Coarse lapilli-tuff with low angle cross-stratification in the Pedro Miguel Formation. (**E**) Fine to very coarse tuff with low angle cross-stratification in the Pedro Miguel Formation. (**F**) Layered fine to medium lapilli-tuff in the Pedro Miguel Formation (Cerro Hodges, position in Fig. 8). (**G**) Coarse lapilli-tuff with amoeboid juvenile clasts in the Pedro Miguel Formation. (**H**) Lens of primary volcanic breccia (with reworked blocks of basalts) in the Pedro Miguel Formation (Cerro Hodges, position in Fig. 8).

Proximal felsic volcanism of the Las Cascadas Formation is followed by a second volcanic phase associated with distal subaerial volcanism in the Culebra, Cucaracha and La Boca Formations between the central Gaillard Cut and the Pacific locks area (Fig. 3). The Culebra Formation marks a marine transgression on top of the Las Cascadas Formation, with transition from lagoon to reef and bathyal environments in respectively the Lower, Emperador and lower Upper Members of the Culebra Formation^11,38,50^. Initial transgression along the Canal is obscured by faults, but core ELCS-5 clearly documents transition from terrestrial to lagoonal environments between the Las Cascadas and Culebra Formations (Fig. 6). Although a large part of the Culebra Formation is composed of marine sediments, there is pervasive occurrence of altered fine to coarse tuffs of intermediate-felsic composition, locally with remnants of lithics, plagioclase and pyroxene. Evidence for such volcanic deposits notably includes bioturbated tuffaceous sediment in the shallow-marine Lower Member (Fig. 7A), and turbidites with bubble-wall shards, planktonic foraminifera and coral fragments in the bathyal lower Upper Member (Fig. 7B). Deposition of these tuffaceous deposits is constrained to ca. 21 to 19 Ma by stratigraphic relationships and previous Sr isotope and U-Pb zircon ages^13,38^ (Fig. 3). A Sr isotope age of 23.07 ± 0.53 Ma in the Lower Member of the Culebra Formation^38^ is inconsistent with new geochronological data and stratigraphic relationships, and so was disregarded in our interpretation. The preceding observations suggest that the marine transgression documented by the Culebra Formation could have been in part synchronous with the end of volcanism of the Las Cascadas volcanic complex. However, finer tuffs and absence of PDC deposit and lava in this formation indicate influence of more distal volcanic centres during most of its deposition.

Marine sedimentation along the Gaillard Cut was followed by a transition to a delta system in the uppermost Culebra Formation^38^, and a range of terrestrial environments in the Cucaracha Formation (i.e., mangroves, coastal swamps, rivers and flood plains)^13,17,38,41^. Minor tuffaceous deposits in the uppermost Culebra Formation record continued distal volcanic activity ca. 19 Ma. In the Cucaracha Formation, continuation of this activity is documented by green to red (more rarely white) clayey paleosols that form most of the unit. In fresh excavations and cores, these paleosols locally preserve an original tuff fabric with flattened ghosts of cm-sized pumices. In addition, the Cucaracha Formation includes a distinctive ignimbrite (5–8 m thick), that is locally found on top of fallout tuff and/or charcoaled tree stumps^13,40,41^ (Fig. 7C). This ignimbrite is an important stratigraphic marker in the Gaillard Cut, and was previously dated at one locality to 18.96 ± 0.90 Ma (Ar-Ar dating) and 18.81 ± 0.30 Ma (U-Pb dating of 2 zircons)^41^. Five new Ar-Ar analyses from this ignimbrite define a better constrained 17.86 ± 0.05 to 17.98 ± 0.05 Ma age of crystallization (Fig. 3, Supplementary Dataset 4). This age means that the Early Miocene terrestrial mammals of the Gaillard Cut (Centenario Fauna) are better correlated to chron C5Dr than C5Er as previously suggested^41^. Geochemical composition of the Cucaracha ignimbrite at different localities is similar to that of previous volcanic products in the Las Cascadas Formation, with possible intermediate to felsic components preserved/mingled in the ignimbrite (Fig. 5). Therefore, consistent with the occurrence of tuffaceous deposits in the Culebra Formation, observations in the Cucaracha Formation support continued distal subaerial volcanism until ca. 18 Ma, with the exceptional emplacement of one ignimbrite.

New excavations and mapping in the Pacific locks area revealed that the Cucaracha Formation likely extends laterally to the south to layered sequences of tuffaceous sandstone with planar bedding to conglomerate. Marine shells and larger benthic foraminifera locally occur in the coarser sandstone and conglomerate. Significantly, a unique 17.90 ± 0.08 Ar-Ar Ma age for a brown intermediate (?) tuff from this unit (Supplementary Dataset 4) indicates at least partly synchronous deposition with the Cucaracha Formation. Therefore, this unit is interpreted to represent the La Boca Formation, as originally recognised by canal geologists based on cores from this area^26,37^. Together, the La Boca and Cucaracha Formations record sedimentation along a palaeo-shoreline during the Early Miocene (ca. 18 Ma), under the influence of continued distal volcanic activity.

### Proximal mafic volcanism (Pedro Miguel Formation)

The third and final phase of volcanism recorded along the Gaillard Cut is associated with the Pedro Miguel Formation which is intermittently exposed between the central Gaillard Cut and the southern termination of the Canal (Fig. 3). Several areas of this unit have previously been mapped along the Gaillard Cut, where they were interpreted as maar-diatreme pyroclastic pipes with large basaltic sills and bedded tuffs^40^. New detailed lithostratigraphic observations of these areas and the Pacific locks exposures provide additional constraints on volcanic processes associated with this unit, showing that it is mostly composed of tuff cones and large mafic intrusions, with only limited evidence for diatremes. Volcanic edifices of the Pedro Miguel Formation are predominantly composed of basaltic to basaltic andesitic tuffs and large mafic intrusions with olivine ± pyroxenes ± plagioclase phenocrysts. These igneous rocks have a similar geochemical signature unique to the Pedro Miguel Formation (Fig. 5). This geochemical signature is also found in numerous dykes and intrusions that crosscut other units of the Gaillard Cut and Pacific locks area. This and new lithostratigraphic observations from Cerro Hodges (see below) clearly indicate that the Pedro Miguel Formation was emplaced after the Bas Obispo, Las Cascadas and Culebra Formations, in stratigraphic continuity to the Cucaracha and La Boca Formations. The emplacement of the Pedro Miguel volcanic deposits temporally overlaps with that of these units (ca. 18 Ma), however its duration remains to be determined. Due to geochemical and age similarities and apparent parental linkage of the tuffs and mafic intrusions, the Pedro Miguel unit is subdivided here into Tuff and Basalt Members (Fig. 3). These members correspond respectively to the original Pedro Miguel and Basalt Formations of the Canal map^26^. However, our regional survey did not confirm the occurrence of the Pedro Miguel Formation east of the Gaillard Cut, suggesting that this unit is restricted to the Canal and possibly eastern Central Panama.

**Figure 8:**
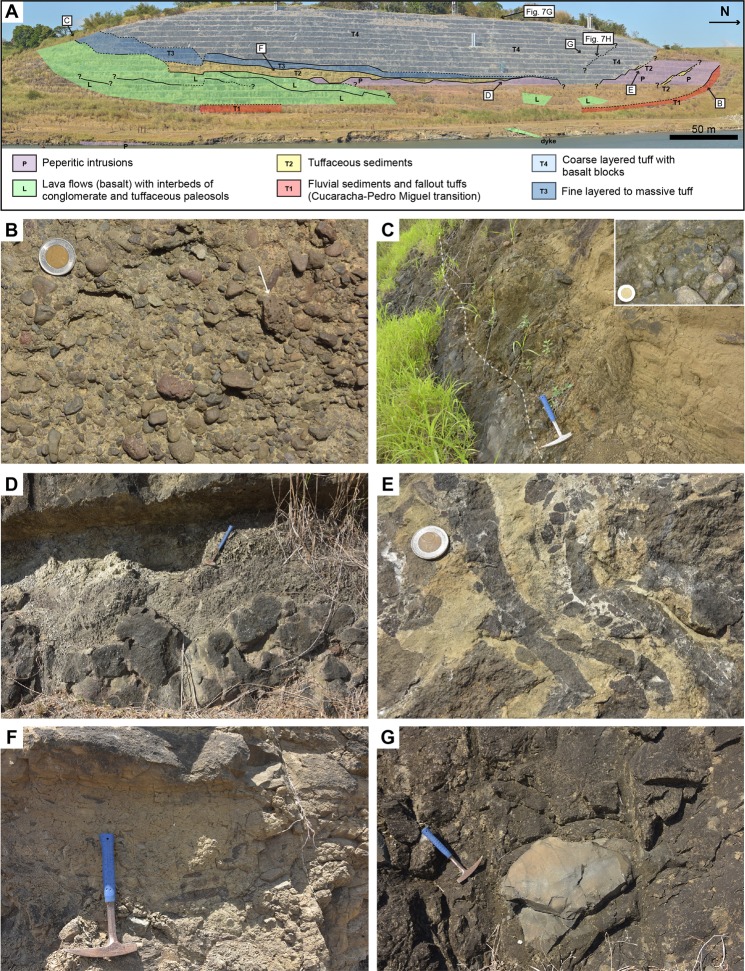
Lithostratigraphic interpretation of the Pedro Miguel Formation at Cerro Hodges (location is given in Fig. 3). (**A**) Overview of lithologies with position of detailed photos. (**B**) Conglomerate in underlying Cucaracha Formation, with scoriaceous clasts (arrow). (**C**) Lava flow overlapped by conglomerate (inset) and layered fine to medium tuff. (**D**) Peperitic intrusion in tuffaceous sediment. (**E**) Peperitic intrusion in tuff. (**F**) Debris flow deposit with reworked fragments of tuff. (**G)** Coarse lapilli-tuff with large block of basalt.

**Figure 9:**
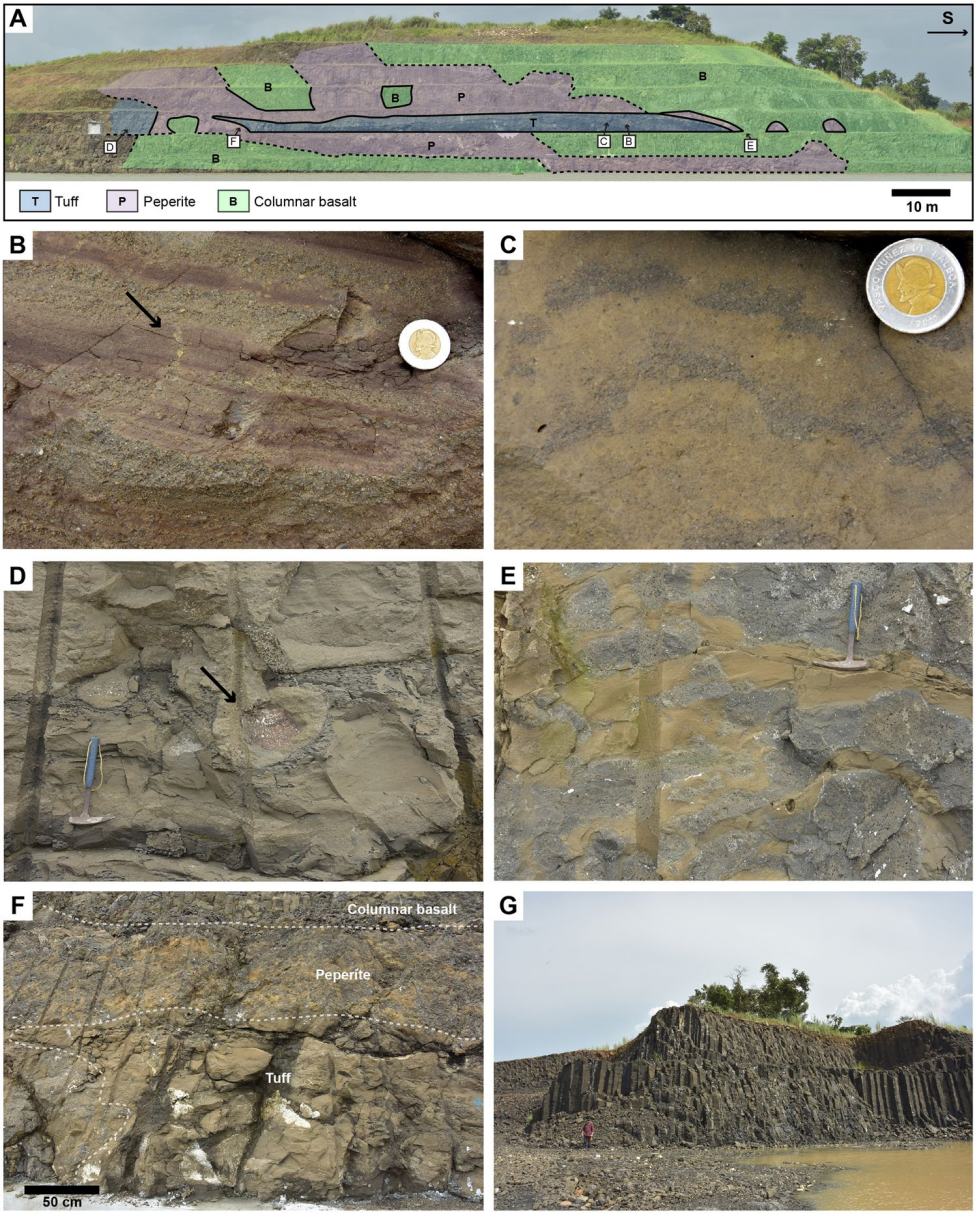
Lithostratigraphic interpretation of the Pedro Miguel Formation at Cerro Fabiana (location is given in Fig. 3). (**A**) Overview of lithologies with position of detailed photos. (**B**) Fine tuff to fine lapilli-tuff with evidence for soft-sedimentation and water escape structure (arrow). (**C**) Fine tuff to medium lapilli-tuff with accretionary lapilli. **(D)** Fine tuff to medium lapilli-tuff with block sag (arrow). (**E**) Peperitic intrusion. (**F**) Relationship between tuff, peperitic contact of basalt sill and interior of the sill with columnar basalt. (**G**) Subvertical columnar jointing in a large basaltic sill (man for scale).

Volcaniclastic deposits of the Pedro Miguel Tuff Member are predominantly composed of fine tuff to primary volcanic breccias with a distinctive layered structure (Figs 7D–H, 8C and 9B). Low angle cross-stratification is common in fine tuff to coarse lapilli-tuff (Fig. 7D,E), whereas the primary volcanic breccias are generally matrix-supported and massive (no grading) (Fig. 7H). Isolated large blocks and bomb sag structures locally occur in the tuff (Figs 8G and 9D). Sorting of the tephra ranges from very poorly sorted to well-sorted. Better sorting is commonly associated with layering at a centimetre scale, which reflects cyclical variations in the tephra size (ash to coarse lapilli), with white zeolite cement filling original pore spaces in the coarser tephra deposits (Fig. 7E,F). Fine to coarse lapilli are predominantly composed of juvenile, amoeboid clasts with a chilled margin (Fig. 7G), with only rare mafic lithics. Small (<0.5 cm) accretionary lapilli are frequent in the finer tuff, but often difficult to recognise due to common alteration of this lithology (Fig. 9C). Accidental clasts in the tuff include larger benthic foraminifera and red algae in the Pacific locks area, suggesting reworking from an underlying shallow-marine (La Boca?) unit. Very rare lava flows are locally interbedded with the tuffs (Fig. 8C). The occurrence of mafic ash, small accretionary lapilli and low angle cross-stratification in the tuffs, with only very rare lavas, are compelling evidence for common phreatomagmatic to rarer strombolian eruption deposits in the Pedro Miguel Formation.

Additional evidence for phreatomagmatic eruptions consists of remarkable, large peperitic intrusions of basalt/basaltic andesite of the Pedro Miguel Basalt Member into the Tuff Member (Figs 8D,E and 9E,F), with a range of ductile to brittle structures typical of interaction of wet material (here tuff) with magma^51^. Similar peperitic textures were observed in some of the Pedro Miguel dykes that crosscut older units of the Gaillard Cut. At Cerro Hodges (Gaillard Cut) and Cerro Fabiana (Pacific locks area), new excavations reveals large scale (100–500 m wide) volcanic structures with mega lenses of tuff embedded in peperitic sills (Figs 8 and 9). The centre of the sills includes coarse basalt with subvertical columnar jointing (Fig. 9G) and very rarely gabbro or dolerite, which grades at the contact with the tuff to highly amygdaloidal basalt and peperite (Fig. 9A,E,F). The wetness of the tuff at the contact with the sills is attested by soft deformation and dewatering structures (9B).

As also suggested by previous work^40^, volcanic evolution of the Pedro Miguel Formation is best exemplified by exposures at Cerro Hodges (Fig. 8). Our lithofacies observations and previous geochemical constraints^40^ show that this sequence records several volcanic events separated by periods of local volcanic quiescence and sedimentation in a terrestrial environment. From bottom to top, this sequence includes: (i) interbedded conglomerate and plant-bearing tuff of the Cucaracha Formation (Fig. 8B); (ii) lava flows of the Pedro Miguel Formation separated by tuffs of undefined origin and basaltic conglomerates (Fig. 8C); (iii) tuffaceous debris flow deposits and sandstone (Fig. 8F); fine layered to massive tuff that include horizons of silicified woods (Fig. 8C); and (iv) coarse layered tuff with large (reworked) blocks of basalts and lenses of basalt breccia (Figs 6G,H and 8G). Although the sequence is intruded by two peperitic sills and affected by pervasive faulting and deformation, our observations suggest that several monogenetic volcanic edifices are superposed in this area. The geometry of the deposits, minor abundance of reworked material in primary volcaniclastic deposits, and the absence of vent-infill (e.g., lacustrine) sediment do not support the occurrence of a diatreme. Instead, these observations suggest the occurrence of superposed tephra cones and possible tuff rings, as also often exposed in other parts of the Pedro Miguel Formation. Prominent hills that are common in this formation (e.g., Cerro Hodges, Cerro Fabiana and Gold Hill, Fig. 2) are erosional remnants that correlate with the occurrence of mafic sills emplaced in older volcanic cones. These topographic characteristics, our regional field work, and new and previous geochemical constraints suggest that the Pedro Miguel Formation, which corresponds to the third and final volcanic phase of the Gaillard Cut, preserves a remarkable example of an extinct monogenetic volcanic field in Central Panama.

## Discussion and Conclusions

**Figure 10:**
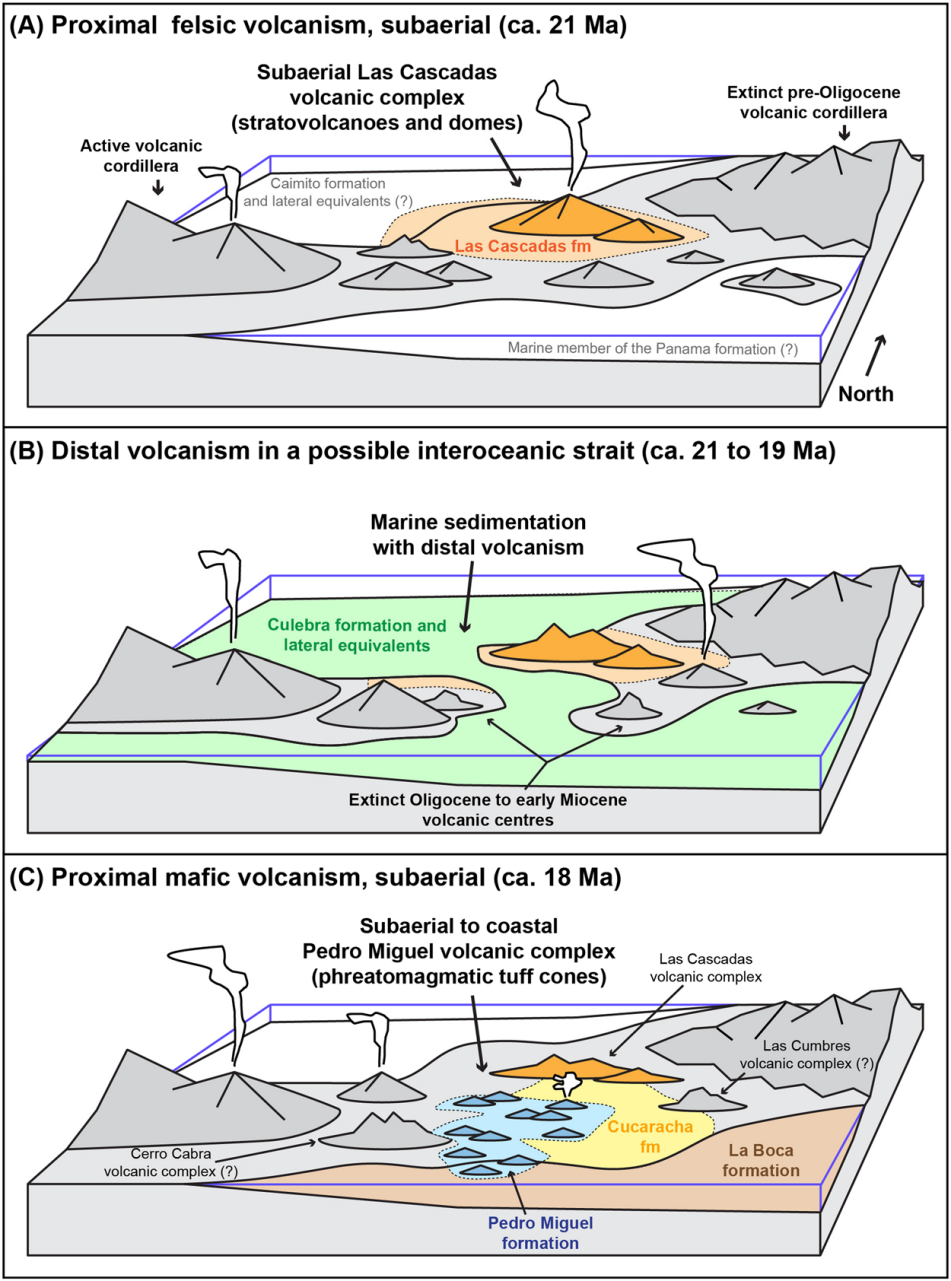
Idealised model of volcanic and palaeo-environmental evolution of Central Panama in the Early Miocene illustrating marine transgression and regression during: (**A**) felsic proximal volcanism of the Las Cascadas volcanic complex in a terrestrial environment (Las Cascadas Formation, ca. 21 Ma); (**B**) distal volcanism in shallow to bathyal marine environments (Culebra Formation, ca. 21 to 19 Ma); and **(C)** proximal mafic (predominantly phreatomagmatic) volcanism of the Pedro Miguel volcanic complex in terrestrial to coastal environments (Cucaracha, La Boca and Pedro Miguel Formations, ca. 18 Ma). Deposition of the Culebra Formation is tentatively associated with the establishment of an ephemeral interoceanic strait ca. 21 to 19 Ma.

The main cause of emergence of the Panama Isthmus is generally considered to be tectonic uplift in response to collision of the Panama volcanic arc (Choco block) with the South American continent (North Andean block)^14,36,44,46,52–55^. However, the timing of this collision and its control on the emergence of the isthmus remain uncertain, as exemplified by proposed ages for initial collision that range from the Late Cretaceous^54^ to the Eocene^14,36^ and Neogene^44,46,53^. In addition, different segments of the Panama volcanic arc have been affected by complex, diachronous uplift events due to local and regional tectonic processes that are not related to collision with South America (e.g., seamount collisions and changing subduction dynamics)^10,12,28,55,56^. Clearly, a large range of tectonic processes could have contributed to the emergence of the Isthmus. In addition, another mechanism of possibly major significance for the emergence of Panama, but which has so far received very limited attention, is magmatic construction of the Panama volcanic arc. Volcanic activity is known to play a critical role in the formation of intraplate oceanic islands, where emergence is controlled by a complex interplay of magmatic, erosional and tectonic processes^57,58^. Similarly, volcanism in Central Panama could also have played a significant role in the emergence of the Panama Isthmus. This volcanism added to previous construction of the main volcanic cordilleras in Panama^34,35^ and could have contributed to obstruction of an interoceanic strait in Central Panama (Fig. 10).

Our results show that volcanic evolution of the Canal area includes two main phases of proximal volcanic activity associated with the formation of stratovolcanoes/composite volcanoes in the Las Cascadas Volcanic Complex (ca. 21 Ma) and superposed monogenetic volcanoes in the Pedro Miguel Volcanic Complex (ca. 18[-?] Ma). Tuffaceous deposits in the Culebra, Cucaracha and La Boca Formation indicate that regional volcanism continued between these two proximal volcanic phases. Similar, albeit more mafic volcanic activity ca. 25 Ma is also attested by new ages from reworked clinopyroxene-phyric andesites in the Bas Obispo Formation and a previous Ar-Ar age^45^ from amphibole-phyric andesites at Cerro Patacon (Fig. 2). Our regional survey suggests that these more distal volcanic phases (relative to the Gaillard Cut) could have been related to the emplacement of distinct, unchartered volcanic complexes in the Las Cumbres and Cerro Cabra areas, which are part of the poorly defined “Panama Formation”^26^ (Fig. 2). Although these possible complexes remain to be investigated in detail, new regional volcano-stratigraphic constraints support the existence of a Central Panama Volcanic Field that formed between at least ca. 25 and 18 Ma. Formation of this field was necessarily associated with development of a volcanic topography during this period. In fact, the geomorphological effects of this volcanism are still evident today in Central Panama with prominent topographic highs often correlating to large volcanic intrusions and intrusive complexes.

As explained above, the geology of the Gaillard Cut shows that Oligocene to Early Miocene volcanism in Central Panama took place during a cycle of marine transgression and regression. This, together with abundant coastal deposits in the Culebra and Cucaracha Formations^17,38^ and lateral transition between the terrestrial Cucaracha and marine La Boca Formations ca. 18 Ma (Fig. 3), suggest that the Gaillard Cut remained very close to a coastal environment during this period. Consistently with new lithofacies observations, this suggests that volcanoes developed over a range of terrestrial to possibly shallow-marine environments (Fig. 10). Although volcanic construction contributed to obstruction of a possible central Panamanian seaway at this time, it is very likely that tectonic processes and/or sea level changes also played a role in controlling palaeo-environmental evolution of Central Panama. Disentangling volcanic and tectonic contributions will require additional work, but our results show that it will be critical to consider volcanic construction to better characterise the palaeo-geographic evolution of Panama and date final emergence of the Isthmus based on direct geological constraints.

## Methods

### Field survey and lithofacies analysis

Our field observations are based on 6 field campaigns between 2015 and 2018 in (i) the Gaillard Cut and Pacific locks area of the Panama Canal (286 localities), and (ii) nearby forested and urbanised areas in Central Panama (452 localities) (Fig. 2). Our survey was designed to provide new, systematic constraints on volcanic processes recorded in new exposures of the Gaillard Cut and Pacific locks area created during widening of the Canal since 2008, as well as their possible lateral equivalents along nearby rivers and roadcuts in Central Panama. This field survey was conducted in collaboration with the Panama Canal Authority that has carried out detailed lithological and structural mapping of the Gaillard Cut and Pacific locks area during ampliation of the Canal. Our study additionally benefited from access to a large collection of cores that were collected during the ampliation of the Canal. Lithological interpretations benefited from microscope petrographic observations of 101 thin sections of volcanic and volcaniclastic rocks from the Gaillard Cut and Pacific locks area.

Although several recent studies benefited from new Canal exposures, there is only one petrological contribution including minor descriptions of volcanic processes^40^, with many areas still lacking significant lithological constraints to reconstruct in detail the volcanic history of the Canal area. Given the scale of the studied area, we do not intend here to provide a complete and detailed description of all our field localities. Instead, we describe and document volcanic and volcaniclastic products with the support of representative field and core photos that provide a novel insight into the diversity of volcanic processes preserved along the Gaillard Cut. Our descriptions followed recent recommendations on volcaniclastic nomenclature^59^.

Due to high structural complexity and common occurrence of small, sub-kilometre sized faulted blocks along the Canal^26^ (Fig. 3), it was not possible to reconstruct a complete lithostratigraphic columns of the studied sequences. Units are generally separated by complex fault systems with variable oblique (strike-slip), normal and inverse components^32^ locally associated with altered slickensided pseudotachylite. However, this intrinsic stratigraphic limitation was addressed based on a synthesis of previous geochronological constraints complemented by 8 new Ar-Ar analysis of volcanic/volcaniclastic rocks (see Ar-Ar dating), 36 new geochemical data to determine spatial extent of igneous units and accurately tie them to our lithofacies observations (see Geochemical analysis) and recognition in the field of new key lithostratigraphic relationships (see Results).

Coordinates of displayed pictures and analysed samples are given in Supplementary Dataset 1.

### Geochemical analysis

Geochemical composition of Oligocene to Miocene volcanic products that are the focus of this geological study have already been analysed by several petrological studies^35,40,44,45^. We complement these data with major and trace element analyses of 35 samples of volcanic and volcaniclastic rocks (lavas, tuffs and dykes) from the Bas Obispo, Las Cascadas, Cucaracha and La Boca Formations, in order to better characterise their stratigraphic extent and relationships. In addition, comparison of these data with field lithofacies observations allowed us to more systematically assess volcanic response to magmatic changes in the studied area.

Whole rock samples were prepared and analysed at the School of Earth and Ocean Sciences at Cardiff University. Samples were first crushed using a steel jaw crusher. An aliquot of approximately 80 ml of the crushed fraction was subsequently milled to a fine powder in an agate planetary ball mill. Approximately 2 g of this fine powder was ignited for two hours in a furnace at 900 °C to remove any volatile substances and determine loss on ignition (LOI) values. Samples were prepared for ICP analysis using the lithium metaborate fusion method. 0.1 ± 0.001 g of each ignited sample was mixed with 0.6 ± 0.004 g of lithium metaborate flux in a platinum crucible. 3–4 drops of lithium iodide wetting agent were added to each mixture which was then fused using the Claisse Fluxy automated fusion system. Each mixture was then dissolved in a 50 ml solution of 20 ml of 10% HNO_3_ and 30 ml of 18.2 Ω deionised water obtained using a Milli-Q purification system. After the mixture had fully dissolved, 1 ml of 100 ppm Rb spike was added to the solution which was then made up to 100 ml with 18.2 Ω deionized water. Approximately 20 ml of each solution was run on the ICP-OES to obtain major element and some trace element abundances. An aliquot of 1 ml of each solution was added to 1 ml of In and Tl and 8 ml of 2% HNO_3_ and run on the ICPMS to obtain trace element abundances. Instruments used to analyse elemental abundances were a Jobin Yvon Horiba Ultima 2 inductively coupled plasma optical emission spectrometer and a Thermo Elemental X7 series inductively-coupled plasma mass spectrometer. Our results are presented in Supplementary Dataset 2 along with standard analyses (JB1a, NIM-N, NIM-P and NM-G).

To ensure consistency across distinct datasets our data were only compared with previous, well-located ICP-MS trace element analyses from the Gaillard Cut^40^. Previous INAA, SEM and XRF trace element data^40^ were not considered due to apparent bias of some key elements between different analytical techniques (e.g., Nb and La/Sm, Supplementary Dataset 3). Sums of major elements previously analysed by INAA^40^ range between 88.98 and 101.13 wt.% (average = 94.60 wt.%, n = 94), which suggest analytical bias and/or intense alteration of the samples (no LOI provided for most of this dataset). LOI in our samples range between 0.87 and 21.02 wt% (average = 6.71 wt.%, n = 35), with the highest LOI found in altered tuffs and igneous dykes. This is consistent with microscope petrographic observations of our samples that show common alteration of volcanic glass and moderate replacement of finer feldspars by secondary clays, with rarer secondary calcite in the most altered samples. Therefore, only immobile trace elements were considered in our interpretations. Given the scope of this study we only present Nb/Y-Zr/Ti^60^, La/Sm-Nb/Th and primitive mantle-normalised multielement diagrams to determine differentiation of analysed igneous rocks (basaltic to dacitic) and to outline main magmatic phases in the studied sequences.

### Ar-Ar dating

Thirteen new ^40^Ar/^39^Ar ages (Supplementary Dataset 4) were obtained for 10 samples by incremental heating methods using the ARGUS-VI mass spectrometer at OSU Argon Geochronology Laboratory. Samples were irradiated for 6 hours (14-OSU-02) in the TRIGA CLICIT nuclear reactor at Oregon State University, along with the FCT sanidine (28.201 ± 0.023 Ma, 1σ) flux monitor^61^. Individual J-values for each sample were calculated by parabolic extrapolation of the measured flux gradient against irradiation height and typically give 0.1–0.2% uncertainties (1σ). The ^40^Ar/^39^Ar incremental heating age determinations were performed on a multi-collector ARGUS-VI mass spectrometer at Oregon State University that has 5 Faraday collectors (all fitted with 1012 Ohm resistors) and 1 ion-counting CuBe electron multiplier (located in a position next to the lowest mass Faraday collector). This allows us to measure simultaneously all argon isotopes, with mass 36 on the multiplier and masses 37 through 40 on the four adjacent Faradays. This configuration provides the advantages of running in a full multi-collector mode while measuring the lowest peak (on mass 36) on the highly sensitive electron multiplier (which has an extremely low dark-noise and a very high peak/noise ratio). Irradiated samples were loaded into Cu-planchettes in an ultra-high vacuum sample chamber and incrementally heated by scanning a defocused 25 W CO2 laser beam in preset patterns across the sample, in order to release the argon evenly. After heating, reactive gases were cleaned up using an SAES Zr-Al ST101 getter operated at 400 °C for several minutes and two SAES Fe-V-Zr ST172 getters operated at 200 °C and room temperature, respectively. All ages were calculated using the corrected decay constant^62^ of 5.530 ± 0.097 × 10−10 1/yr (2σ) as reported by Min *et al*.^63^. Incremental heating plateau ages and isochron ages were calculated as weighted means with 1/σ2 as weighting factor and as YORK2 least-square fits with correlated errors using the ArArCALC v2.7.2 software^64^ available from the http://earthref.org/ArArCALC/ website.

## Supplementary information

Supplementary Dataset 1

Supplementary Dataset 2

Supplementary Dataset 3

Supplementary Dataset 4
