# Supplementary material for: Volcanic contribution to emergence of Central Panama in the Early Miocene
Source: Sci Rep. 2019 Feb 5;9:1417. doi: 10.1038/s41598-018-37790-2 (PMC6363779; doi:10.1038/s41598-018-37790-2)
Supplement: Supplementary file 1 — Supplementary Dataset 1 [file 41598_2018_37790_MOESM1_ESM.pdf]

Volcanic contribution to emergence of Central Panama in the early Miocene

David M. Buchs[1,2] (buchsd@cardiff.ac.uk), Derek Irving[3], Henry Coombs[1], Roberto Miranda[3], Jian Wang[1], Maurylis Coronado[3], Rodrigo Arrocha[3], Mauricio Lacerda[3], Creed Goff[3], Eladio Almengor[3], Enier Portugal[3], Pastora Franceschi[3], Eric Chichaco[4], Stewart D. Redwood[5]

[1]*School of Earth and Ocean Sciences, Cardiff University, UK*
[2]*Smithsonian Tropical Research Institute, Panama*
[3]*Ingineering Division, Panama Canal Authority, Panama*
[4]*Instituto de Geociencias, University of Panama, Panama*
[5]*Independant consulting geologist, Panama*

Supplementary File 1 - Location of pictures

	Latitude (°)	Longitude (°)
**Figure 4a**	9.105921	-79.693397
**Figure 4b**	9.100217	-79.688500
**Figure 4c**	9.080650	-79.675100
**Figure 4d**	9.074683	-79.672733
**Figure 4e**	9.056700	-79.661117
**Figure 4f**	9.073239	-79.672605
**Figure 4g**	9.071517	-79.674283
**Figure 4h**	9.061747	-79.666083
**Figure 7a**	9.075167	-79.675634
**Figure 7b**	9.080649	-79.678026
**Figure 7c**	9.031500	-79.636767
**Figure 7d**	9.030418	-79.638999
**Figure 7e**	9.019600	-79.619167
**Figure 7f**	9.041789	-79.644857
**Figure 7g**	Cerro Hodges, shown in Figure 8	
**Figure 7h**	Cerro Hodges, shown in Figure 8	
