# Supplementary material for: Volcanic contribution to emergence of Central Panama in the Early Miocene
Source: Sci Rep. 2019 Feb 5;9:1417. doi: 10.1038/s41598-018-37790-2 (PMC6363779; doi:10.1038/s41598-018-37790-2)

Volcanic contribution to emergence of Central Panama in the early Miocene

David M. Buchs[1,2] (buchsd@cardiff.ac.uk), Derek Irving[3], Henry Coombs[1], Roberto Miranda[3], Jian Wang[1], Maurylis Coronado[3], Rodrigo Arrocha[3], Mauricio Lacerda[3], Creed Goff[3], Eladio Almengor[3], Enier Portugal[3], Pastora Franceschi[3], Eric Chichaco[4], Stewart D. Redwood[5]

[1]*School of Earth and Ocean Sciences, Cardiff University, UK*
[2]*Smithsonian Tropical Research Institute, Panama*
[3]*Ingineering Division, Panama Canal Authority, Panama*
[4]*Instituto de Geociencias, University of Panama, Panama*
[5]*Independant consulting geologist, Panama*

Supplementary File 4 - Analytical uncertainty (Farris et al.)

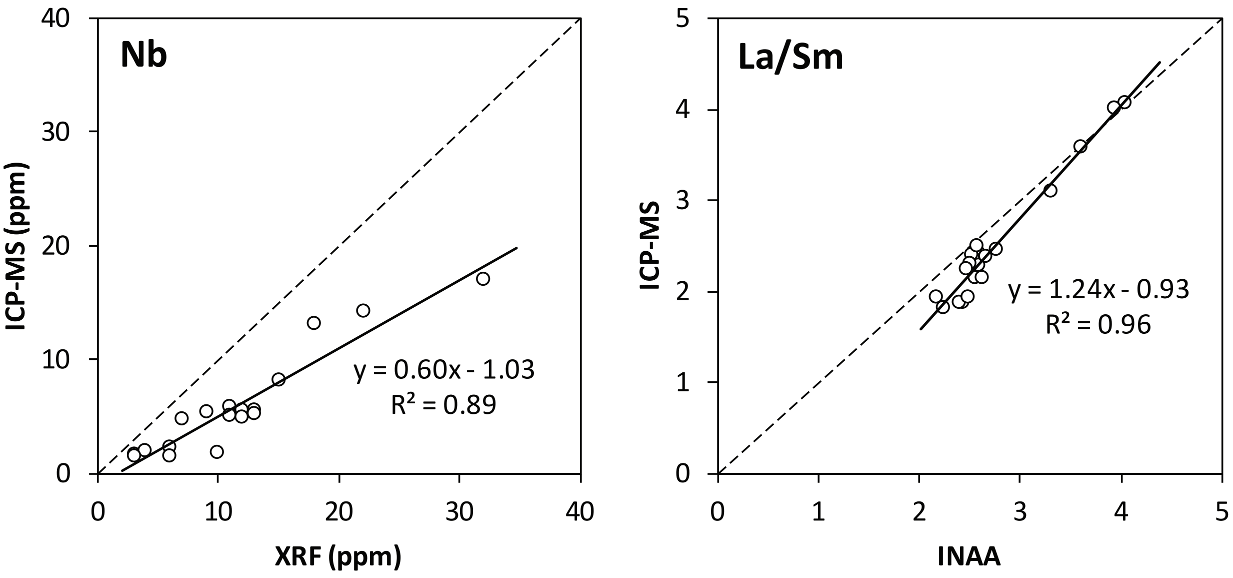

Supplement: Supplementary file 3 — Supplementary Dataset 3 [file 41598_2018_37790_MOESM3_ESM.pdf]
