# Supplementary material for: Volcanic contribution to emergence of Central Panama in the Early Miocene
Source: Sci Rep. 2019 Feb 5;9:1417. doi: 10.1038/s41598-018-37790-2 (PMC6363779; doi:10.1038/s41598-018-37790-2)

Volcanic contribution to emergence of Central Panama in the early Miocene

David M. Buchs[1,2] (buchsd@cardiff.ac.uk), Derek Irving[3], Henry Coombs[1], Roberto Miranda[3], Jian Wang[1], Maurylis Coronado[3], Rodrigo Arrocha[3], Mauricio Lacerda[3], Creed Goff[3], Eladio Almengor[3], Enier Portugal[3], Pastora Franceschi[3], Eric Chichaco[4], Stewart D. Redwood[5]

[1]*School of Earth and Ocean Sciences, Cardiff University, UK*
[2]*Smithsonian Tropical Research Institute, Panama*
[3]*Ingineering Division, Panama Canal Authority, Panama*
[4]*Instituto de Geociencias, University of Panama, Panama*
[5]*Independant consulting geologist, Panama*

Supplementary File 2 - Ar/Ar dating

						Plateau ages (2σ)		
Sample	Formation	Lithology	Latitude	Longitude	Locality	Plagioclase	Groundmass	Preferred age
DAT-11	La Boca	tuff	8.987458	-79.596966	New Pacific locks	17.90 ± 0.08 Ma	-	17.90 ± 0.08 Ma
DAT-19	Cucaracha	dacitic ignimbrite	9.006743	-79.608079	New Pacific locks	17.92 ± 0.05 Ma	-	17.95 ± 0.05 Ma
DAT-24	Cucaracha	dacitic ignimbrite	9.016570	-79.620190	New Pacific locks	17.86 ± 0.05 Ma	-	17.82 ± 0.05 Ma
DAT-26	Cucaracha	dacitic ignimbrite	9.007189	-79.610127	New Pacific locks	17.96 ± 0.06 Ma	-	17.96 ± 0.06 Ma
D16-002	Cucaracha	dacitic ignimbrite	9.047755	-79.653597	Hodges Hill	17.98 ± 0.05 Ma	18.76 ± 0.06 Ma	17.98 ± 0.05 Ma
D16-080	Cucaracha	dacitic ignimbrite	9.039270	-79.646191	Model Slope	17.93 ± 0.05 Ma	-	17.93 ± 0.05 Ma
D16-020	Las Cascadas	dacitic ignimbrite	9.070815	-79.673861	Cunette	20.91 ± 0.06 Ma	19.55 ± 0.05 Ma	20.91 ± 0.06 Ma
D16-101	Las Cascadas	dacite lava flow	9.068894	-79.669564	La Pita	21.05 ± 0.06 Ma	21.00 ± 0.03 Ma	21.05 ± 0.06 Ma
D16-007	Bas Obispo	andesite clast	9.105927	-79.693470	Chagres Crossing	-	24.66 ± 0.11 Ma	24.66 ± 0.11 Ma
D16-009	Bas Obispo	andesite clast	9.105927	-79.693470	Chagres Crossing	25.61 ± 0.20 Ma	-	25.61 ± 0.20 Ma

DAT-11 > La Boca Fm. > Plagioclase
PANAMA | IRVING (15-16)
16-OSU-03 (3C48-16) > Incremental Heating > Dan Miggins

Information on Analysis and Constants Used in Calculations

Sample = **DAT-11**
Material = **Plagioclase**
Location = **La Boca Fm.**
Analyst = **Dan Miggins**
Project = **PANAMA | IRVING (15-16)**
Mass Discrimination Law = **LIN**
Irradiation = **16-OSU-03 (3C48-16)**
J = **0.00146779 ± 0.00000151**
FCT-NM = **28.201 ± 0.023 Ma**
IGSN = Undefined
Preferred Age = Undefined
Classification = Undefined
Experiment Type = **Incremental Heating**
Extraction Method = Undefined
Instrument = **ARGUS-VI-D**
Lithology = Undefined
Lat-Lon = Undefined - Undefined
Collector Calibrations = **36Ar**
Age Equations = **Min et al. (2000)**
Negative Intensities = **Allowed**
Decay 40K = **5.530 ± 0.048 E-10 1/a**
Decay 39Ar = **2.940 ± 0.016 E-07 1/h**
Decay 37Ar = **8.230 ± 0.012 E-04 1/h**
Decay 36Cl = **2.257 ± 0.015 E-06 1/a**
Decay 40K(EC,β⁺) = **0.580 ± 0.009 E-10 1/a**
Decay 40K(β⁻) = **4.950 ± 0.043 E-10 1/a**
Atmospheric 40/36(a) = **295.50**
Atmospheric 38/36(a) = **0.1869**
Production 39/37(ca) = **0.000676 ± 0.000009**
Production 38/37(ca) = **0.000072 ± 0.000009**
Production 36/37(ca) = **0.000266 ± 0.000000**
Production 40/39(k) = **0.003823 ± 0.000102**
Scaling Ratio K/Ca = **0.430**
Abundance Ratio 40K/K = **1.1700 ± 0.0100 E-04**
Atomic Weight K = **39.0983 ± 0.0001 g**

Results	40(a)/36(a) ± 2σ	40(r)/39(k) ± 2σ	Age ± 2σ (Ma)	MSWD	39Ar(k) (%,n)	K/Ca ± 2σ
**Age Plateau**<br>Error Mean		6.77738 ± 0.02780<br>± 0.41%	**17.90 ± 0.08**<br>**± 0.46%**	2.69<br>0%	83.51<br>14	0.0306 ± 0.0001
			Full External Error ± 0.41	1.78	2σ Confidence Limit	
			Analytical Error ± 0.07	1.6395	Error Magnification	
**Total Fusion Age**		6.59797 ± 0.06727<br>± 1.02%	**17.43 ± 0.18**<br>**± 1.04%**		25	0.0307 ± 0.0001
			Full External Error ± 0.43			
			Analytical Error ± 0.18			
**Normal Isochron**<br>Error Chron	294.46 ± 3.62<br>± 1.23%	6.78843 ± 0.05087<br>± 0.75%	**17.93 ± 0.14**<br>**± 0.77%**	2.82<br>0%	83.51<br>14	
			Full External Error ± 0.43	1.82	2σ Confidence Limit	
			Analytical Error ± 0.13	1.6788	Error Magnification	
**Inverse Isochron**<br>Error Chron	294.55 ± 3.63<br>± 1.23%	6.78869 ± 0.05107<br>± 0.75%	**17.93 ± 0.14**<br>**± 0.78%**	2.85<br>0%	83.51<br>14	
			Full External Error ± 0.43	1.82	2σ Confidence Limit	
				0.0000116378	Convergence	
				45%	Spreading Factor	

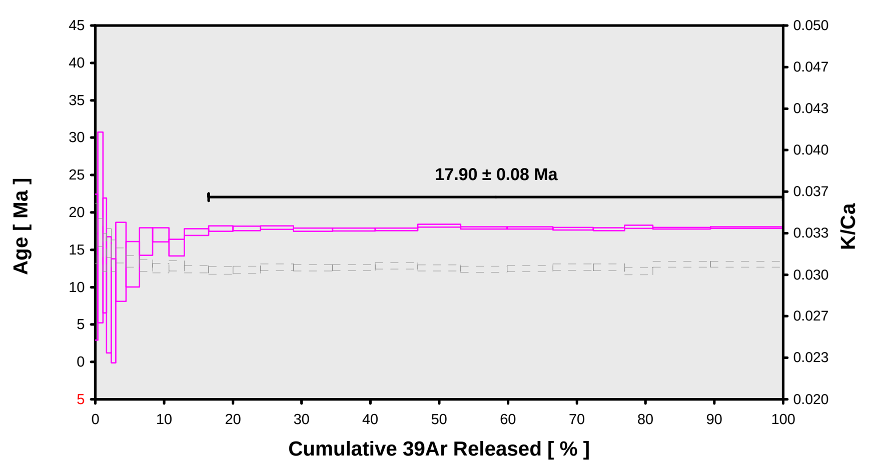

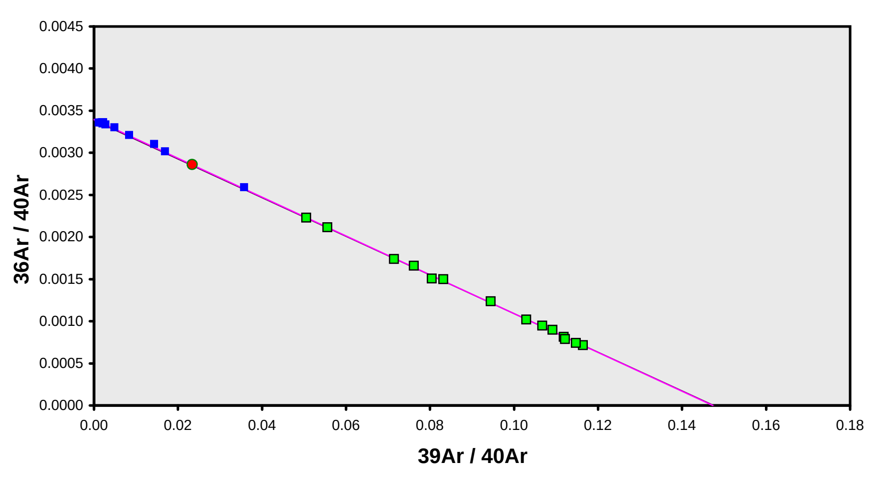

DAT-19 > Panama Canal > Plagioclase
PANAMA | IRVING (15-16)
16-OSU-01 (1A40-16) > Incremental Heating > Dan Miggins

Information on Analysis and Constants Used in Calculations

Sample = **DAT-19**
Material = **Plagioclase**
Location = **Panama Canal**
Analyst = **Dan Miggins**
Project = **PANAMA | IRVING (15-16)**
Mass Discrimination Law = **LIN**
Irradiation = **16-OSU-01 (1A40-16)**
J = **0.00152327 ± 0.00000154**
FCT-NM = **28.201 ± 0.023 Ma**
IGSN = Undefined
Preferred Age = Undefined
Classification = Undefined
Experiment Type = **Incremental Heating**
Extraction Method = Undefined
Instrument = **ARGUS-VI-D**
Lithology = Undefined
Lat-Lon = Undefined - Undefined
Collector Calibrations = **36Ar**
Age Equations = **Min et al. (2000)**
Negative Intensities = **Allowed**
Decay 40K = **5.530 ± 0.048 E-10 1/a**
Decay 39Ar = **2.940 ± 0.016 E-07 1/h**
Decay 37Ar = **8.230 ± 0.012 E-04 1/h**
Decay 36Cl = **2.257 ± 0.015 E-06 1/a**
Decay 40K(EC,β^+) = **0.580 ± 0.009 E-10 1/a**
Decay 40K(β^-) = **4.950 ± 0.043 E-10 1/a**
Atmospheric 40/36(a) = **295.50**
Atmospheric 38/36(a) = **0.1869**
Production 39/37(ca) = **0.000676 ± 0.000009**
Production 38/37(ca) = **0.000072 ± 0.000009**
Production 36/37(ca) = **0.000266 ± 0.000000**
Production 40/39(k) = **0.003823 ± 0.000102**
Scaling Ratio K/Ca = **0.430**
Abundance Ratio 40K/K = **1.1700 ± 0.0100 E-04**
Atomic Weight K = **39.0983 ± 0.0001 g**

Results	40(a)/36(a) ± 2σ		40(r)/39(k) ± 2σ		Age ± 2σ (Ma)		MSWD	39Ar(k) (%,n)	K/Ca ± 2σ
**Age Plateau** Error Mean			6.53759	± 0.00993<br>± 0.15%	**17.92**	**± 0.05**<br>**± 0.25%**	1.57<br>3%	97.96<br>28	0.0303 ± 0.0001
					Full External Error	± 0.41	1.54	2σ Confidence Limit	
					Analytical Error	± 0.03	1.2517	Error Magnification	
**Total Fusion Age**			6.53771	± 0.00821<br>± 0.13%	**17.92**	**± 0.04**<br>**± 0.24%**		29	0.0303 ± 0.0000
					Full External Error	± 0.41			
					Analytical Error	± 0.02			
**Normal Isochron** Error Chron	300.18	± 11.34<br>± 3.78%	6.54376	± 0.01324<br>± 0.20%	**17.94**	**± 0.05**<br>**± 0.28%**	1.96<br>0%	97.96<br>28	
					Full External Error	± 0.41	1.55	2σ Confidence Limit	
					Analytical Error	± 0.04	1.4018	Error Magnification	
**Inverse Isochron**	309.38	± 8.76<br>± 2.83%	6.52786	± 0.01065<br>± 0.16%	**17.89**	**± 0.05**<br>**± 0.26%**	1.15<br>27%	97.96<br>28	
					Full External Error	± 0.41	1.55	2σ Confidence Limit	
							0.0000500493	Convergence	
							17%	Spreading Factor	

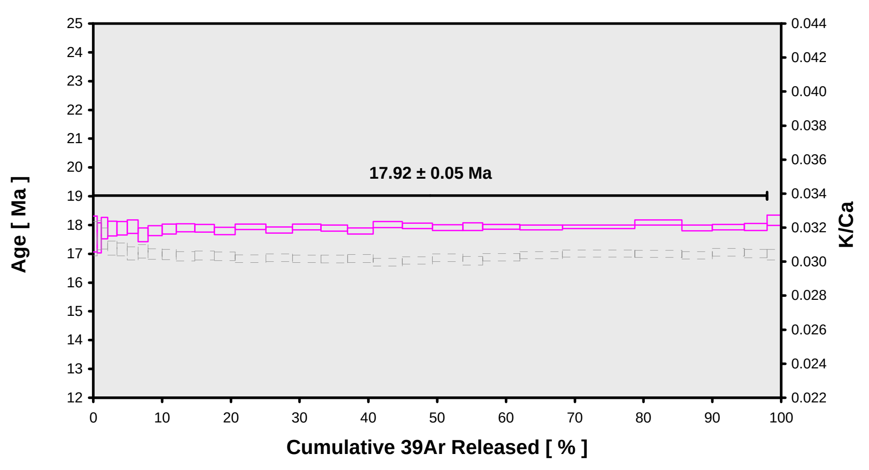

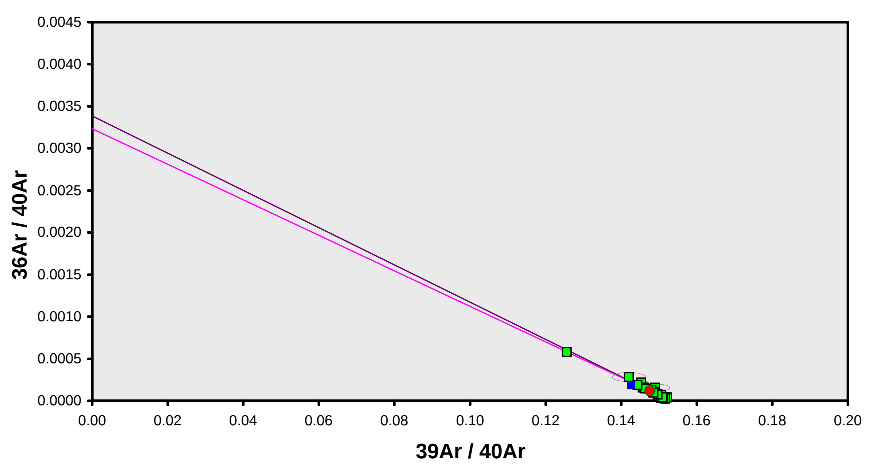

DAT-24 > Panama Canal > Plagioclase
PANAMA | IRVING (15-16)
16-OSU-01 (1A41-16) > Incremental Heating > Dan Miggins

Information on Analysis and Constants Used in Calculations

Sample = **DAT-24**
Material = **Plagioclase**
Location = **Panama Canal**
Analyst = **Dan Miggins**
Project = **PANAMA | IRVING (15-16)**
Mass Discrimination Law = **LIN**
Irradiation = **16-OSU-01 (1A41-16)**
J = **0.00151620 ± 0.00000152**
FCT-NM = **28.201 ± 0.023 Ma**
IGSN = Undefined
Preferred Age = Undefined
Classification = Undefined
Experiment Type = **Incremental Heating**
Extraction Method = Undefined
Instrument = **ARGUS-VI-D**
Lithology = Undefined
Lat-Lon = Undefined - Undefined
Collector Calibrations = **36Ar**
Age Equations = **Min et al. (2000)**
Negative Intensities = **Allowed**
Decay 40K = **5.530 ± 0.048 E-10 1/a**
Decay 39Ar = **2.940 ± 0.016 E-07 1/h**
Decay 37Ar = **8.230 ± 0.012 E-04 1/h**
Decay 36Cl = **2.257 ± 0.015 E-06 1/a**
Decay 40K(EC,β⁺) = **0.580 ± 0.009 E-10 1/a**
Decay 40K(β⁻) = **4.950 ± 0.043 E-10 1/a**
Atmospheric 40/36(a) = **295.50**
Atmospheric 38/36(a) = **0.1869**
Production 39/37(ca) = **0.000676 ± 0.000009**
Production 38/37(ca) = **0.000072 ± 0.000009**
Production 36/37(ca) = **0.000266 ± 0.000000**
Production 40/39(k) = **0.003823 ± 0.000102**
Scaling Ratio K/Ca = **0.430**
Abundance Ratio 40K/K = **1.1700 ± 0.0100 E-04**
Atomic Weight K = **39.0983 ± 0.0001 g**

Results	40(a)/36(a) ± 2σ		40(r)/39(k) ± 2σ		Age ± 2σ (Ma)		MSWD	39Ar(k) (%,n)	K/Ca ± 2σ
**Age Plateau** Error Mean			6.54695	± 0.01141<br>± 0.17%	**17.86**	**± 0.05**<br>**± 0.26%**	1.86<br>0%	100.00<br>29	0.0304 ± 0.0001
					Full External Error	± 0.40	1.53	2σ Confidence Limit	
					Analytical Error	± 0.03	1.3645	Error Magnification	
**Total Fusion Age**			6.54615	± 0.00871<br>± 0.13%	**17.86**	**± 0.04**<br>**± 0.24%**		29	0.0304 ± 0.0001
					Full External Error	± 0.40			
					Analytical Error	± 0.02			
**Normal Isochron** Error Chron	298.77	± 9.74<br>± 3.26%	6.53912	± 0.01844<br>± 0.28%	**17.84**	**± 0.06**<br>**± 0.34%**	1.91<br>0%	100.00<br>29	
					Full External Error	± 0.41	1.54	2σ Confidence Limit	
					Analytical Error	± 0.05	1.3835	Error Magnification	
**Inverse Isochron** Error Chron	298.31	± 9.71<br>± 3.26%	6.54307	± 0.01834<br>± 0.28%	**17.85**	**± 0.06**<br>**± 0.34%**	1.90<br>0%	100.00<br>29	
					Full External Error	± 0.41	1.54	2σ Confidence Limit	
							0.0000122185	Convergence	
							21%	Spreading Factor	

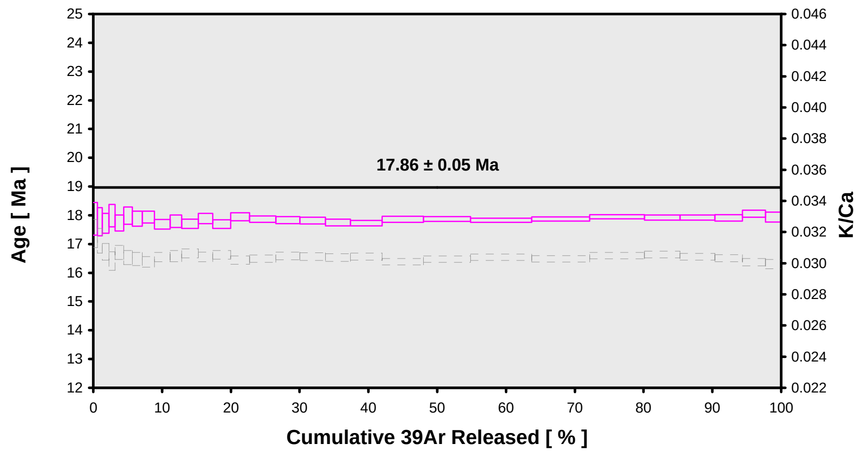

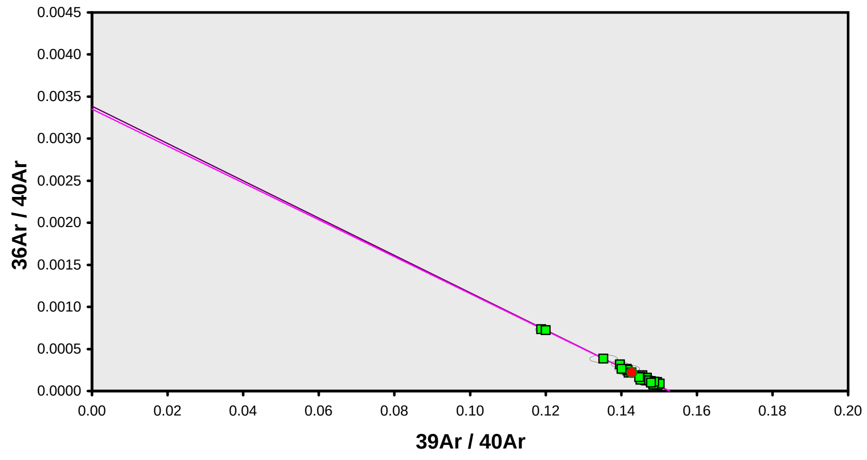

DAT-26 > Panama Canal > Plagioclase
PANAMA | IRVING (15-16)
16-OSU-03 (3C47-16) > Incremental Heating > Dan Miggins

Information on Analysis and Constants Used in Calculations

Sample = **DAT-26**
Material = **Plagioclase**
Location = **Panama Canal**
Analyst = **Dan Miggins**
Project = **PANAMA | IRVING (15-16)**
Mass Discrimination Law = **LIN**
Irradiation = **16-OSU-03 (3C47-16)**
J = **0.00147513 ± 0.00000153**
FCT-NM = **28.201 ± 0.023 Ma**
IGSN = Undefined
Preferred Age = Undefined
Classification = Undefined
Experiment Type = **Incremental Heating**
Extraction Method = Undefined
Instrument = **ARGUS-VI-D**
Lithology = Undefined
Lat-Lon = Undefined - Undefined
Collector Calibrations = **36Ar**
Age Equations = **Min et al. (2000)**
Negative Intensities = **Allowed**
Decay 40K = **5.530 ± 0.048 E-10 1/a**
Decay 39Ar = **2.940 ± 0.016 E-07 1/h**
Decay 37Ar = **8.230 ± 0.012 E-04 1/h**
Decay 36Cl = **2.257 ± 0.015 E-06 1/a**
Decay 40K(EC,β^+) = **0.580 ± 0.009 E-10 1/a**
Decay 40K(β^-) = **4.950 ± 0.043 E-10 1/a**
Atmospheric 40/36(a) = **295.50**
Atmospheric 38/36(a) = **0.1869**
Production 39/37(ca) = **0.000676 ± 0.000009**
Production 38/37(ca) = **0.000072 ± 0.000009**
Production 36/37(ca) = **0.000266 ± 0.000000**
Production 40/39(k) = **0.003823 ± 0.000102**
Scaling Ratio K/Ca = **0.430**
Abundance Ratio 40K/K = **1.1700 ± 0.0100 E-04**
Atomic Weight K = **39.0983 ± 0.0001 g**

Results

Results	40(a)/36(a) ± 2σ	40(r)/39(k) ± 2σ	Age ± 2σ (Ma)	MSWD	39Ar(k) (%,n)	K/Ca ± 2σ
**Age Plateau**		6.76515 ± 0.01546<br>± 0.23%	**17.96 ± 0.06<br>± 0.31%**	1.13<br>34%	69.01<br>10	0.0309 ± 0.0001
			Full External Error ± 0.41	1.94	2σ Confidence Limit	
			Analytical Error ± 0.04	1.0640	Error Magnification	
**Total Fusion Age**		6.74040 ± 0.01942<br>± 0.29%	**17.89 ± 0.06<br>± 0.35%**		25	0.0292 ± 0.0001
			Full External Error ± 0.41			
			Analytical Error ± 0.05			
**Normal Isochron**	299.26 ± 6.35<br>± 2.12%	6.72887 ± 0.06252<br>± 0.93%	**17.86 ± 0.17<br>± 0.95%**	1.08<br>38%	69.01<br>10	
			Full External Error ± 0.44	2.00	2σ Confidence Limit	
			Analytical Error ± 0.17	1.0381	Error Magnification	
**Inverse Isochron**	299.25 ± 6.35<br>± 2.12%	6.72937 ± 0.06240<br>± 0.93%	**17.86 ± 0.17<br>± 0.95%**	1.08<br>37%	69.01<br>10	
			Full External Error ± 0.44	2.00	2σ Confidence Limit	
				0.0000377366	Convergence	
				17%	Spreading Factor	

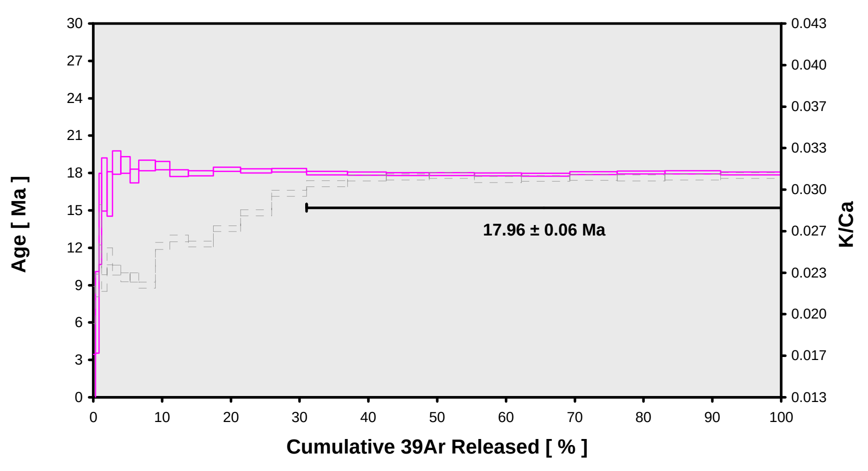

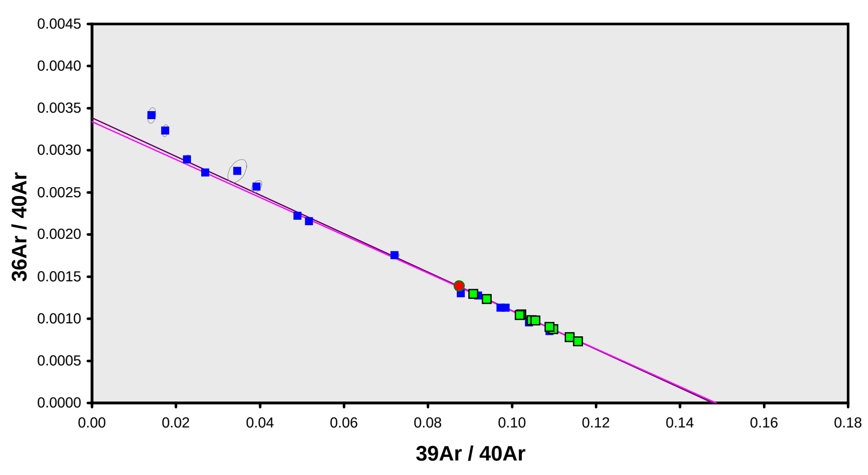

EXP#17D31977 > D16-002 > Plagioclase > IRVING (17-21) PANAMA > PANAMA CANAL 17-OSU-06 (6F12-17) > Incremental Heating > Dan Miggins

Information on Analysis and Constants Used in Calculations

Project = **IRVING (17-21)**
Sample = **D16-002**
Material = **Plagioclase**
Location = **Panama Canal**
Region = **Panama**
Analyst = **Dan Miggins**
Irradiation = **17-OSU-06 (6F12-17)**
Position = **X: 0 | Y: 0 | Z/H: 15.72955 mm**
FCT-NM Age = **28.201 ± 0.023 Ma**
FCT-NM Reference = **Kuiper et al (2008)**
FCT-NM 40Ar/39Ar Ratio = **9.60146 ± 0.01018**
FCT-NM J-value = **0.00163698 ± 0.00000174**
Air Shot 40Ar/36Ar = **303.2270 ± 0.4427**
Air Shot MDF = **0.99362113 ± 0.00067905 (LIN)**
Experiment Type = **Incremental Heating**
Extraction Method = **Bulk Laser Heating**
Heating = **64 sec**
Isolation = **6.00 min**
Instrument = **ARGUS-VI-D**
Preferred Age = **Plateau Age**
Age Classification = **Crystallization Age**
IGSN = Undefined
Rock Class = Undefined
Lithology = Undefined
Lat-Lon = Undefined - Undefined
Age Equations = **Min et al. (2000)**
Negative Intensities = **Allowed**
Collector Calibrations = **36Ar**
Decay 40K = **5.530 ± 0.048 E-10 1/a**
Decay 39Ar = **2.940 ± 0.016 E-07 1/h**
Decay 37Ar = **8.230 ± 0.012 E-04 1/h**
Decay 36Cl = **2.257 ± 0.015 E-06 1/a**
Decay 40K(EC,β^+) = **0.580 ± 0.009 E-10 1/a**
Decay 40K(β^-) = **4.950 ± 0.043 E-10 1/a**
Atmospheric 40/36(a) = **295.50**
Atmospheric 38/36(a) = **0.1869**
Production 39/37(ca) = **0.0006425 ± 0.0000059**
Production 38/37(ca) = **0.0001800 ± 0.0000173**
Production 36/37(ca) = **0.0002703 ± 0.0000005**
Production 40/39(k) = **0.000607 ± 0.000059**
Production 38/39(k) = **0.012077 ± 0.000011**
Production 36/38(cl) = **262.80 ± 1.71**
Scaling Ratio K/Ca = **0.430**
Abundance Ratio 40K/K = **1.1700 ± 0.0100 E-04**
Atomic Weight K = **39.0983 ± 0.0001 g**

Nice Plateau

Results

Results	40(a)/36(a) ± 2σ		40(r)/39(k) ± 2σ		Age ± 2σ (Ma)		MSWD	39Ar(k) (%,n)	K/Ca ± 2σ
**Age Plateau**			6.10424	± 0.01023<br>± 0.17%	**17.98**	**± 0.05<br>± 0.27%**	0.98<br>49%	97.94<br>21	0.0302 ± 0.0002
				Full External Error		± 0.41	1.63	2σ Confidence Limit	
				Analytical Error		± 0.03	1.0000	Error Magnification	
**Total Fusion Age**			6.10685	± 0.01774<br>± 0.29%	**17.99**	**± 0.06<br>± 0.36%**		24	0.0302 ± 0.0001
				Full External Error		± 0.41			
				Analytical Error		± 0.05			
**Normal Isochron**	**295.48**	**± 1.40<br>± 0.47%**	6.10361	± 0.01362<br>± 0.22%	**17.98**	**± 0.06<br>± 0.31%**	1.03<br>42%	97.94<br>21	
				Full External Error		± 0.41	1.65	2σ Confidence Limit	
				Analytical Error		± 0.04	1.0145	Error Magnification	
**Inverse Isochron**	**295.43**	**± 1.40<br>± 0.47%**	6.10473	± 0.01364<br>± 0.22%	**17.98**	**± 0.06<br>± 0.31%**	1.03<br>42%	97.94<br>21	
				Full External Error		± 0.41	1.65	2σ Confidence Limit	
				Analytical Error		± 0.04	1.0143	Error Magnification	
							76%	Spreading Factor	

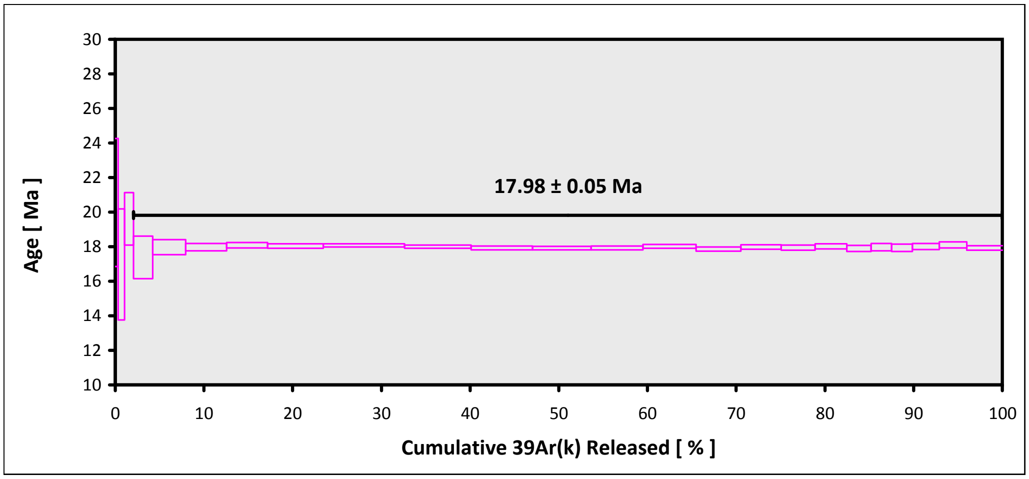

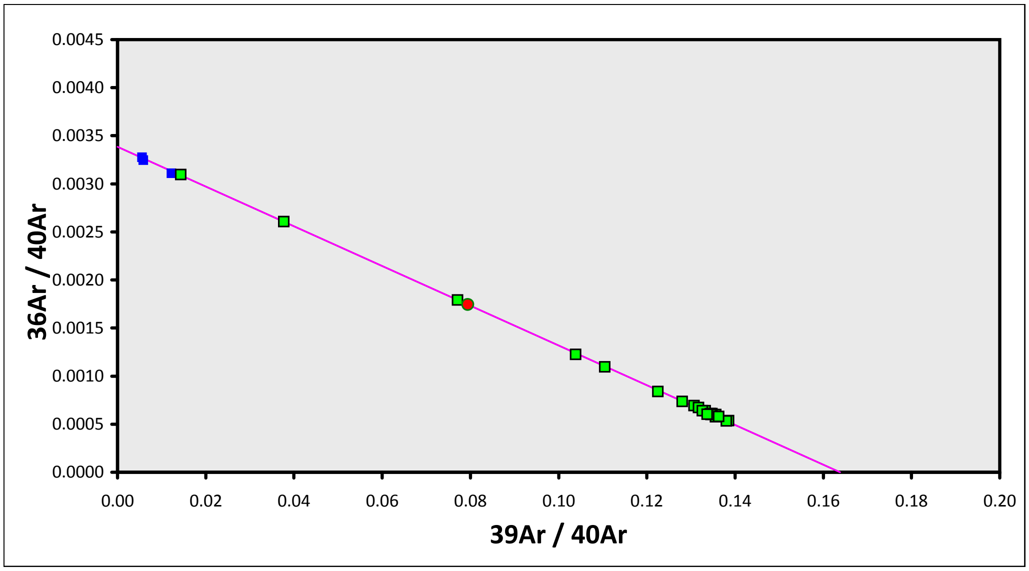

EXP#17D32416 > D16-002 > Groundmass > IRVING (17-21)
PANAMA > PANAMA CANAL
17-OSU-06 (6X19-17) > Incremental Heating > Dan Miggins

Information on Analysis and Constants Used in Calculations

Project = **IRVING (17-21)**
Sample = **D16-002**
Material = **Groundmass**
Location = **Panama Canal**
Region = **Panama**
Analyst = **Dan Miggins**
Irradiation = **17-OSU-06 (6X19-17)**
Position = **X: 0 | Y: 0 | Z/H: 21.88121 mm**
FCT-NM Age = **28.201 ± 0.023 Ma**
FCT-NM Reference = **Kuiper et al (2008)**
FCT-NM 40Ar/39Ar Ratio = **9.63313 ± 0.00617**
FCT-NM J-value = **0.00163160 ± 0.00000104**
Air Shot 40Ar/36Ar = **303.2080 ± 0.4093**
Air Shot MDF = **0.99363641 ± 0.00066541 (LIN)**
Experiment Type = **Incremental Heating**
Extraction Method = **Bulk Laser Heating**
Heating = **64 sec**
Isolation = **6.00 min**
Instrument = **ARGUS-VI-D**
Preferred Age = **Plateau Age**
Age Classification = **Crystallization Age**
IGSN = Undefined
Rock Class = Undefined
Lithology = Undefined
Lat-Lon = Undefined - Undefined
Age Equations = **Min et al. (2000)**
Negative Intensities = **Allowed**
Collector Calibrations = **36Ar**
Decay 40K = **5.530 ± 0.048 E-10 1/a**
Decay 39Ar = **2.940 ± 0.016 E-07 1/h**
Decay 37Ar = **8.230 ± 0.012 E-04 1/h**
Decay 36Cl = **2.257 ± 0.015 E-06 1/a**
Decay 40K(EC,β^+) = **0.580 ± 0.009 E-10 1/a**
Decay 40K(β^-) = **4.950 ± 0.043 E-10 1/a**
Atmospheric 40/36(a) = **295.50 ± 0.70**
Atmospheric 38/36(a) = **0.1869**
Production 39/37(ca) = **0.0006425 ± 0.0000059**
Production 38/37(ca) = **0.0001800 ± 0.0000173**
Production 36/37(ca) = **0.0002703 ± 0.0000005**
Production 40/39(k) = **0.000607 ± 0.000059**
Production 38/39(k) = **0.012077 ± 0.000011**
Production 36/38(cl) = **262.80 ± 1.71**
Scaling Ratio K/Ca = **0.430**
Abundance Ratio 40K/K = **1.1700 ± 0.0100 E-04**
Atomic Weight K = **39.0983 ± 0.0001 g**

Plateau, but disturbed age spectrum

Results

Results	40(a)/36(a) ± 2σ		40(r)/39(k) ± 2σ		Age ± 2σ (Ma)		MSWD	39Ar(k) (%,n)	K/Ca ± 2σ
**Age Plateau**			6.39039	± 0.01945<br>± 0.30%	**18.76**	**± 0.06<br>± 0.33%**	1.50<br>19%	55.13<br>6	1.14 ± 0.04
					Full External Error	± 0.43	2.26	2σ Confidence Limit	
					Analytical Error	± 0.06	1.2249	Error Magnification	
**Total Fusion Age**			6.45484	± 0.01385<br>± 0.21%	**18.95**	**± 0.05<br>± 0.25%**		31	0.99 ± 0.01
					Full External Error	± 0.43			
					Analytical Error	± 0.04			
**Normal Isochron**	**296.71**	**± 2.61<br>± 0.88%**	6.37147	± 0.04511<br>± 0.71%	**18.70**	**± 0.13<br>± 0.72%**	2.41<br>5%	55.13<br>6	
					Full External Error	± 0.44	2.41	2σ Confidence Limit	
					Analytical Error	± 0.13	1.5509	Error Magnification	
**Inverse Isochron**	**296.72**	**± 2.62<br>± 0.88%**	6.37155	± 0.04516<br>± 0.71%	**18.70**	**± 0.13<br>± 0.72%**	2.41<br>5%	55.13<br>6	
					Full External Error	± 0.44	2.41	2σ Confidence Limit	
					Analytical Error	± 0.13	1.5526	Error Magnification	
							34%	Spreading Factor	

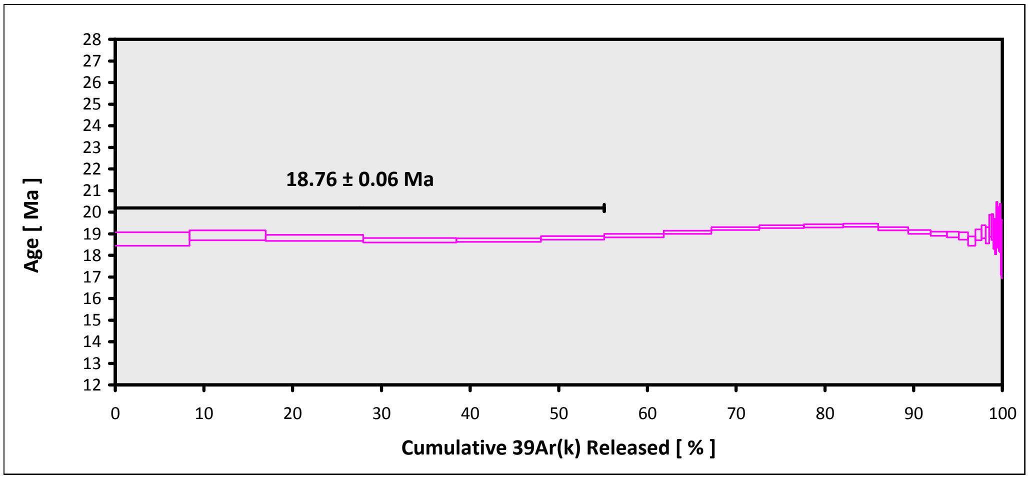

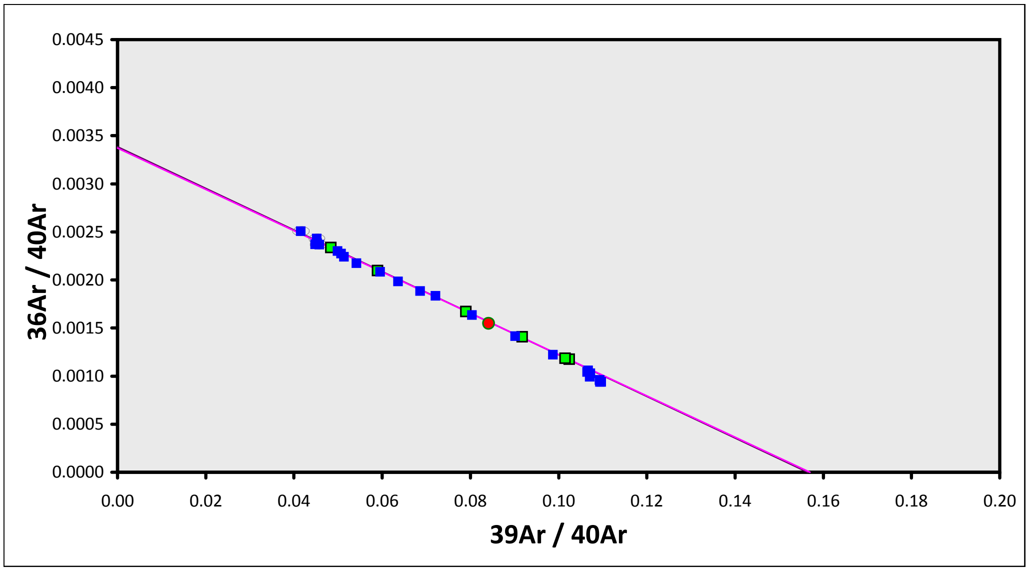

EXP#17D32084 > D16-080 > Plagioclase > IRVING (17-21) PANAMA > PANAMA CANAL 17-OSU-06 (6F9-17) > Incremental Heating > Dan Miggins

Information on Analysis and Constants Used in Calculations

Project = **IRVING (17-21)**
Sample = **D16-080**
Material = **Plagioclase**
Location = **Panama Canal**
Region = **Panama**
Analyst = **Dan Miggins**
Irradiation = **17-OSU-06 (6F9-17)**
Position = **X: 0 | Y: 0 | Z/H: 11.54186 mm**
FCT-NM Age = **28.201 ± 0.023 Ma**
FCT-NM Reference = **Kuiper et al (2008)**
FCT-NM 40Ar/39Ar Ratio = **9.59059 ± 0.01026**
FCT-NM J-value = **0.00163883 ± 0.00000175**
Air Shot 40Ar/36Ar = **302.9460 ± 0.4272**
Air Shot MDF = **0.99384740 ± 0.00067335 (LIN)**
Experiment Type = **Incremental Heating**
Extraction Method = **Bulk Laser Heating**
Heating = **64 sec**
Isolation = **6.00 min**
Instrument = **ARGUS-VI-D**
Preferred Age = **Plateau Age**
Age Classification = **Crystallization Age**
IGSN = Undefined
Rock Class = Undefined
Lithology = Undefined
Lat-Lon = Undefined - Undefined
Age Equations = **Min et al. (2000)**
Negative Intensities = **Allowed**
Collector Calibrations = **36Ar**
Decay 40K = **5.530 ± 0.048 E-10 1/a**
Decay 39Ar = **2.940 ± 0.016 E-07 1/h**
Decay 37Ar = **8.230 ± 0.012 E-04 1/h**
Decay 36Cl = **2.257 ± 0.015 E-06 1/a**
Decay 40K(EC,β^+) = **0.580 ± 0.009 E-10 1/a**
Decay 40K(β^-) = **4.950 ± 0.043 E-10 1/a**
Atmospheric 40/36(a) = **295.50**
Atmospheric 38/36(a) = **0.1869**
Production 39/37(ca) = **0.0006425 ± 0.0000059**
Production 38/37(ca) = **0.0001800 ± 0.0000173**
Production 36/37(ca) = **0.0002703 ± 0.0000005**
Production 40/39(k) = **0.000607 ± 0.000059**
Production 38/39(k) = **0.012077 ± 0.000011**
Production 36/38(cl) = **262.80 ± 1.71**
Scaling Ratio K/Ca = **0.430**
Abundance Ratio 40K/K = **1.1700 ± 0.0100 E-04**
Atomic Weight K = **39.0983 ± 0.0001 g**

Nice Plateau

Results

Results	40(a)/36(a) ± 2σ		40(r)/39(k) ± 2σ		Age ± 2σ (Ma)		MSWD	39Ar(k) (%,n)	K/Ca ± 2σ
**Age Plateau**			6.07994	± 0.01325	**17.93**	**± 0.05**	1.70	94.91	0.0310 ± 0.0002
Error Mean				± 0.22%		**± 0.30%**	3%	20	
				Full External Error		± 0.41	1.65	2σ Confidence Limit	
				Analytical Error		± 0.04	1.3050	Error Magnification	
**Total Fusion Age**			6.08380	± 0.01416	**17.94**	**± 0.06**		24	0.0310 ± 0.0001
				± 0.23%		**± 0.31%**			
				Full External Error		± 0.41			
				Analytical Error		± 0.04			
**Normal Isochron**	**299.15**	**± 2.86**	6.07017	± 0.01464	**17.90**	**± 0.06**	1.23	94.91	
		**± 0.96%**		± 0.24%		**± 0.32%**	22%	20	
				Full External Error		± 0.41	1.67	2σ Confidence Limit	
				Analytical Error		± 0.04	1.1108	Error Magnification	
**Inverse Isochron**	**299.73**	**± 2.80**	6.06639	± 0.01433	**17.89**	**± 0.06**	1.18	94.91	
		**± 0.93%**		± 0.24%		**± 0.32%**	26%	20	
				Full External Error		± 0.41	1.67	2σ Confidence Limit	
				Analytical Error		± 0.04	1.0879	Error Magnification	
							55%	Spreading Factor	

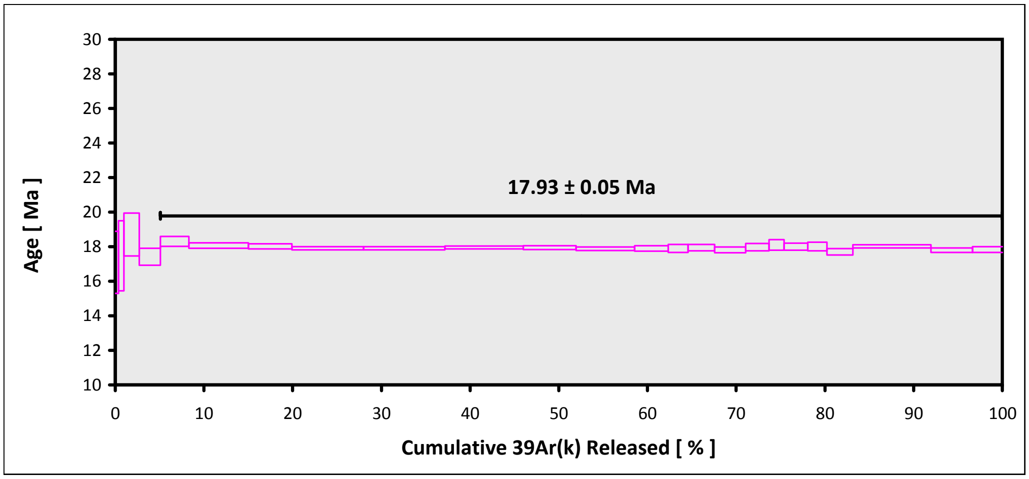

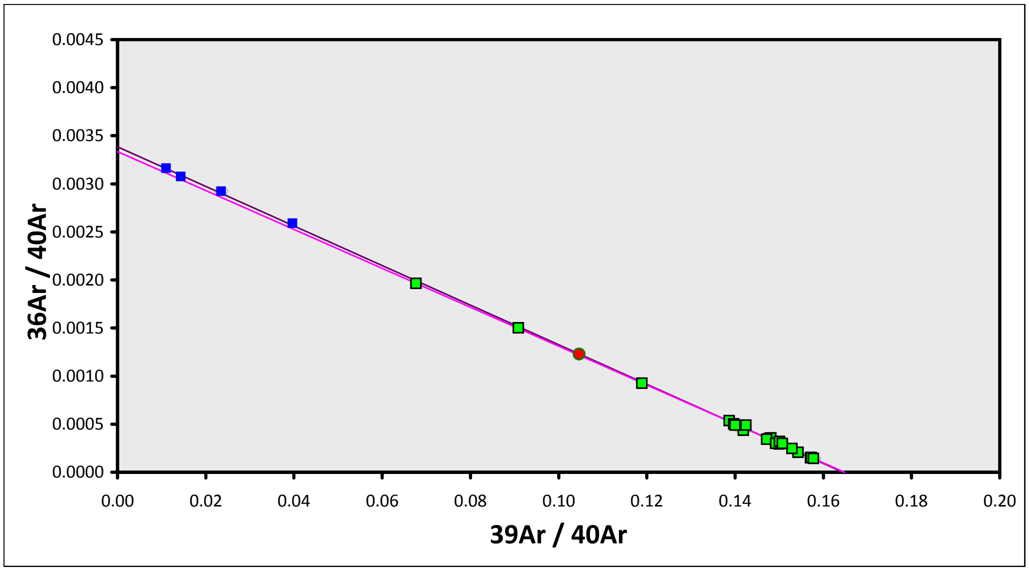

EXP#17D32834 > D16-080 > Groundmass > IRVING (17-21)
PANAMA > PANAMA CANAL
17-OSU-06 (6X24-17) > Incremental Heating > Dan Miggins

Information on Analysis and Constants Used in Calculations

Project = **IRVING (17-21)**
Sample = **D16-080**
Material = **Groundmass**
Location = **Panama Canal**
Region = **Panama**
Analyst = **Dan Miggins**
Irradiation = **17-OSU-06 (6X24-17)**
Position = **X: 0 | Y: 0 | Z/H: 29.47502 mm**
FCT-NM Age = **28.201 ± 0.023 Ma**
FCT-NM Reference = **Kuiper et al (2008)**
FCT-NM 40Ar/39Ar Ratio = **9.69648 ± 0.00611**
FCT-NM J-value = **0.00162094 ± 0.00000102**
Air Shot 40Ar/36Ar = **302.1960 ± 0.3989**
Air Shot MDF = **0.99445339 ± 0.00066403 (LIN)**
Experiment Type = **Incremental Heating**
Extraction Method = **Bulk Laser Heating**
Heating = **64 sec**
Isolation = **6.00 min**
Instrument = **ARGUS-VI-D**
Preferred Age = **Total Fusion**
Age Classification = **Crystallization Age**
IGSN = Undefined
Rock Class = Undefined
Lithology = Undefined
Lat-Lon = Undefined - Undefined
Age Equations = **Min et al. (2000)**
Negative Intensities = **Allowed**
Collector Calibrations = **36Ar**
Decay 40K = **5.530 ± 0.048 E-10 1/a**
Decay 39Ar = **2.940 ± 0.016 E-07 1/h**
Decay 37Ar = **8.230 ± 0.012 E-04 1/h**
Decay 36Cl = **2.257 ± 0.015 E-06 1/a**
Decay 40K(EC,β^+) = **0.580 ± 0.009 E-10 1/a**
Decay 40K(β^-) = **4.950 ± 0.043 E-10 1/a**
Atmospheric 40/36(a) = **295.50**
Atmospheric 38/36(a) = **0.1869**
Production 39/37(ca) = **0.0006425 ± 0.0000059**
Production 38/37(ca) = **0.0001800 ± 0.0000173**
Production 36/37(ca) = **0.0002703 ± 0.0000005**
Production 40/39(k) = **0.000607 ± 0.000059**
Production 38/39(k) = **0.012077 ± 0.000011**
Production 36/38(cl) = **262.80 ± 1.71**
Scaling Ratio K/Ca = **0.430**
Abundance Ratio 40K/K = **1.1700 ± 0.0100 E-04**
Atomic Weight K = **39.0983 ± 0.0001 g**

Significant 39Ar recoil

Results

Results	40(a)/36(a) ± 2σ	40(r)/39(k) ± 2σ	Age ± 2σ (Ma)	MSWD	39Ar(k) (%,n)	K/Ca ± 2σ
**Age Plateau**<br>Cannot Calculate						
**Total Fusion Age**		6.32317 ± 0.00290 ± 0.05%	**18.44 ± 0.02 ± 0.13%**<br>Full External Error ± 0.42<br>Analytical Error ± 0.01		31	1.15 ± 0.00
**Normal Isochron**<br>Cannot Calculate						
**Inverse Isochron**<br>Cannot Calculate						

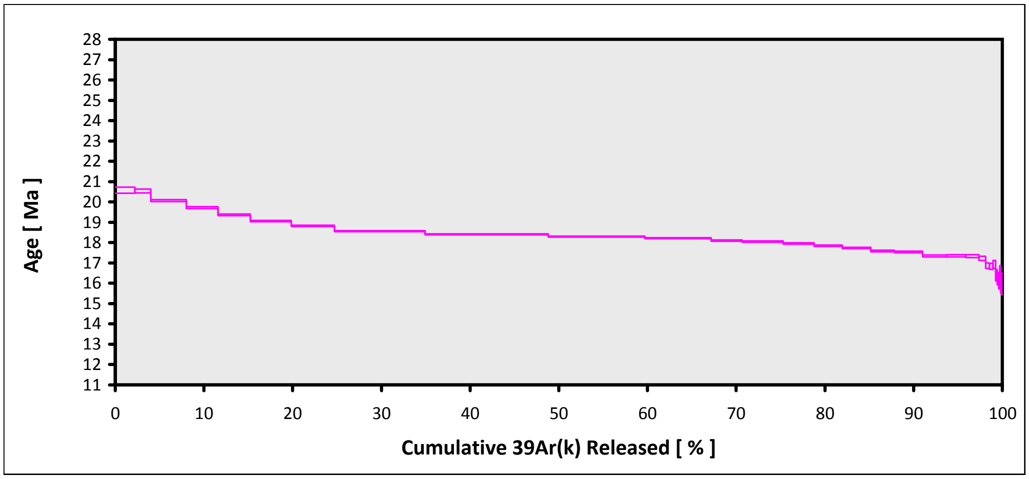

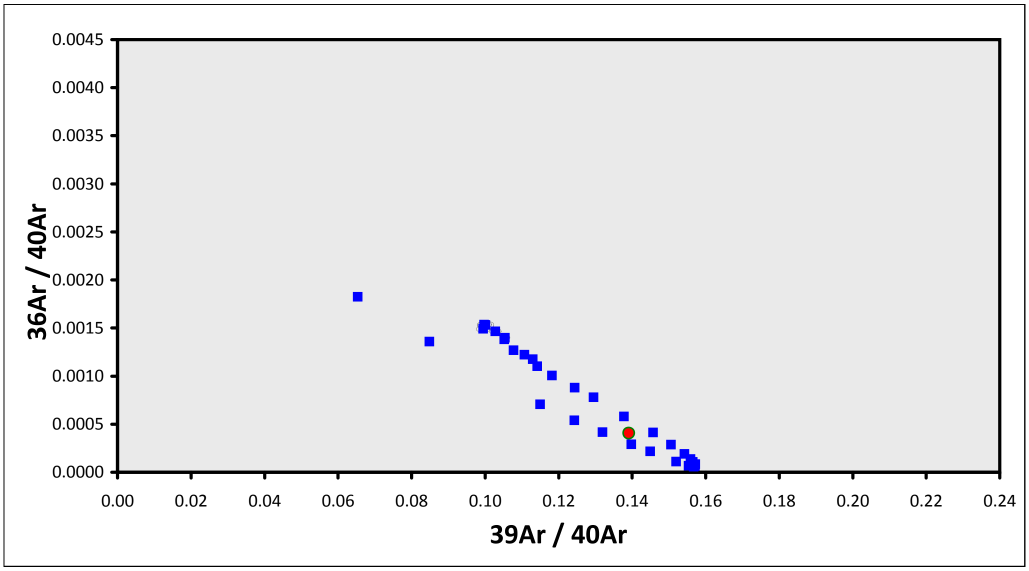

EXP#17D31870 > D16-020 > Plagioclase > IRVING (17-21)
PANAMA > PANAMA CANAL
17-OSU-06 (6F10-17) > Incremental Heating > Dan Miggins

Information on Analysis and Constants Used in Calculations

Project = **IRVING (17-21)**
Sample = **D16-020**
Material = **Plagioclase**
Location = **Panama Canal**
Region = **Panama**
Analyst = **Dan Miggins**
Irradiation = **17-OSU-06 (6F10-17)**
Position = **X: 0 | Y: 0 | Z/H: 12.81908 mm**
FCT-NM Age = **28.201 ± 0.023 Ma**
FCT-NM Reference = **Kuiper et al (2008)**
FCT-NM 40Ar/39Ar Ratio = **9.59303 ± 0.01026**
FCT-NM J-value = **0.00163842 ± 0.00000175**
Air Shot 40Ar/36Ar = **303.1500 ± 0.4426**
Air Shot MDF = **0.99368309 ± 0.00067922 (LIN)**
Experiment Type = **Incremental Heating**
Extraction Method = **Bulk Laser Heating**
Heating = **64 sec**
Isolation = **6.00 min**
Instrument = **ARGUS-VI-D**
Preferred Age = **Plateau Age**
Age Classification = **Crystallization Age**
IGSN = Undefined
Rock Class = Undefined
Lithology = Undefined
Lat-Lon = Undefined - Undefined
Age Equations = **Min et al. (2000)**
Negative Intensities = **Allowed**
Collector Calibrations = **36Ar**
Decay 40K = **5.530 ± 0.048 E-10 1/a**
Decay 39Ar = **2.940 ± 0.016 E-07 1/h**
Decay 37Ar = **8.230 ± 0.012 E-04 1/h**
Decay 36Cl = **2.257 ± 0.015 E-06 1/a**
Decay 40K(EC,β^+) = **0.580 ± 0.009 E-10 1/a**
Decay 40K(β^-) = **4.950 ± 0.043 E-10 1/a**
Atmospheric 40/36(a) = **295.50**
Atmospheric 38/36(a) = **0.1869**
Production 39/37(ca) = **0.0006425 ± 0.0000059**
Production 38/37(ca) = **0.0001800 ± 0.0000173**
Production 36/37(ca) = **0.0002703 ± 0.0000005**
Production 40/39(k) = **0.000607 ± 0.000059**
Production 38/39(k) = **0.012077 ± 0.000011**
Production 36/38(cl) = **262.80 ± 1.71**
Scaling Ratio K/Ca = **0.430**
Abundance Ratio 40K/K = **1.1700 ± 0.0100 E-04**
Atomic Weight K = **39.0983 ± 0.0001 g**

Nice Plateau

Results

Results	40(a)/36(a) ± 2σ		40(r)/39(k) ± 2σ		Age ± 2σ (Ma)		MSWD	39Ar(k) (%,n)	K/Ca ± 2σ
**Age Plateau**			7.09982	± 0.01213	**20.91**	**± 0.06**	1.70	97.96	0.0339 ± 0.0002
Error Mean				± 0.17%		**± 0.27%**	2%	22	
				Full External Error		± 0.47	1.62	2σ Confidence Limit	
				Analytical Error		± 0.04	1.3026	Error Magnification	
**Total Fusion Age**			7.09515	± 0.01120	**20.90**	**± 0.06**		24	0.0339 ± 0.0001
				± 0.16%		**± 0.26%**			
				Full External Error		± 0.47			
				Analytical Error		± 0.03			
**Normal Isochron**	**292.93**	**± 2.73**	7.11083	± 0.01436	**20.95**	**± 0.06**	1.64	97.96	
No Convergence		**± 0.93%**		± 0.20%		**± 0.29%**	4%	22	
				Full External Error		± 0.48	1.63	2σ Confidence Limit	
				Analytical Error		± 0.04	1.2815	Error Magnification	
**Inverse Isochron**	**293.63**	**± 2.73**	7.10555	± 0.01442	**20.93**	**± 0.06**	1.64	97.96	
Error Chron		**± 0.93%**		± 0.20%		**± 0.29%**	4%	22	
				Full External Error		± 0.48	1.63	2σ Confidence Limit	
				Analytical Error		± 0.04	1.2809	Error Magnification	
							66%	Spreading Factor	

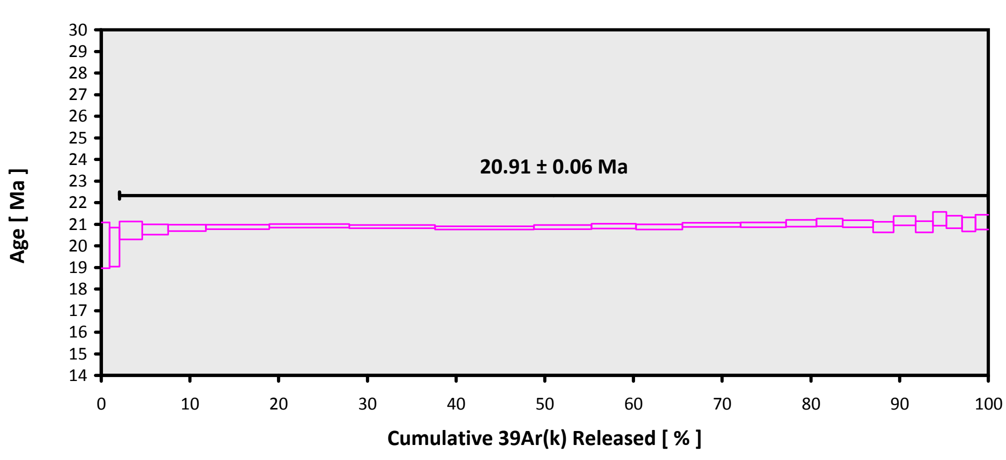

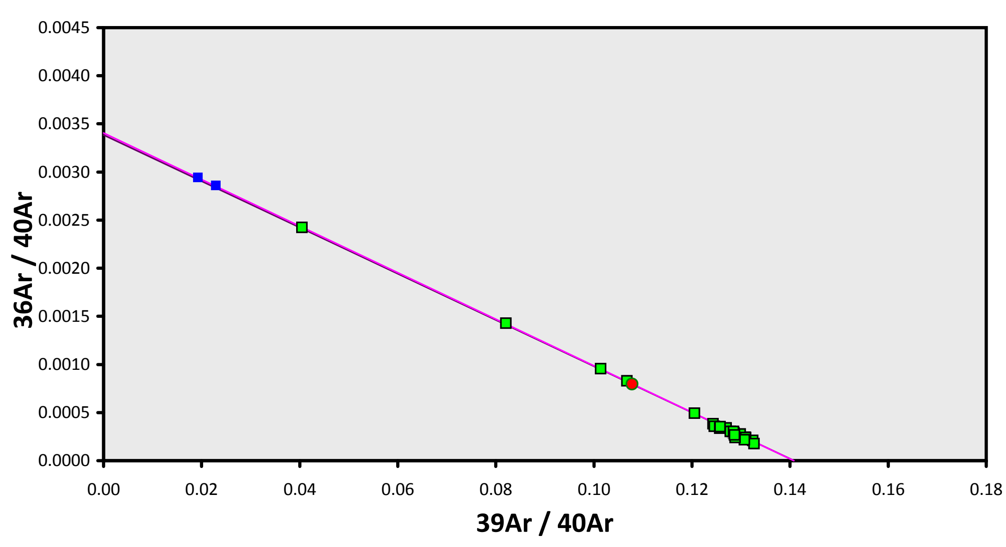

EXP#17D32467 > D16-020 > Groundmass > IRVING (17-21)
PANAMA > PANAMA CANAL
17-OSU-06 (6X20-17) > Incremental Heating > Dan Miggins

Information on Analysis and Constants Used in Calculations

Project = **IRVING (17-21)**
Sample = **D16-020**
Material = **Groundmass**
Location = **Panama Canal**
Region = **Panama**
Analyst = **Dan Miggins**
Irradiation = **17-OSU-06 (6X20-17)**
Position = **X: 0 | Y: 0 | Z/H: 23.58538 mm**
FCT-NM Age = **28.201 ± 0.023 Ma**
FCT-NM Reference = **Kuiper et al (2008)**
FCT-NM 40Ar/39Ar Ratio = **9.64522 ± 0.00608**
FCT-NM J-value = **0.00162955 ± 0.00000103**
Air Shot 40Ar/36Ar = **302.9320 ± 0.1727**
Air Shot MDF = **0.99385868 ± 0.00059521 (LIN)**
Experiment Type = **Incremental Heating**
Extraction Method = **Bulk Laser Heating**
Heating = **64 sec**
Isolation = **6.00 min**
Instrument = **ARGUS-VI-D**
Preferred Age = **Mini Plateau**
Age Classification = **Crystallization Age**
IGSN = Undefined
Rock Class = Undefined
Lithology = Undefined
Lat-Lon = Undefined - Undefined
Age Equations = **Min et al. (2000)**
Negative Intensities = **Allowed**
Collector Calibrations = **36Ar**
Decay 40K = **5.530 ± 0.048 E-10 1/a**
Decay 39Ar = **2.940 ± 0.016 E-07 1/h**
Decay 37Ar = **8.230 ± 0.012 E-04 1/h**
Decay 36Cl = **2.257 ± 0.015 E-06 1/a**
Decay 40K(EC,β^+) = **0.580 ± 0.009 E-10 1/a**
Decay 40K(β^-) = **4.950 ± 0.043 E-10 1/a**
Atmospheric 40/36(a) = **295.50 ± 0.70**
Atmospheric 38/36(a) = **0.1869**
Production 39/37(ca) = **0.0006425 ± 0.0000059**
Production 38/37(ca) = **0.0001800 ± 0.0000173**
Production 36/37(ca) = **0.0002703 ± 0.0000005**
Production 40/39(k) = **0.000607 ± 0.000059**
Production 38/39(k) = **0.012077 ± 0.000011**
Production 36/38(cl) = **262.80 ± 1.71**
Scaling Ratio K/Ca = **0.430**
Abundance Ratio 40K/K = **1.1700 ± 0.0100 E-04**
Atomic Weight K = **39.0983 ± 0.0001 g**

Mini Plateau...argon loss Considered Minimum Age

Results

Results	40(a)/36(a) ± 2σ		40(r)/39(k) ± 2σ		Age ± 2σ (Ma)		MSWD	39Ar(k) (%,n)	K/Ca ± 2σ
**Age Plateau**			6.67210	± 0.01463	**19.55**	**± 0.05**	12.42	30.18	2.39 ± 0.06
Error Mean				± 0.22%		**± 0.25%**	0%	4	
					Full External Error ± 0.44		2.63	2σ Confidence Limit	
					Analytical Error ± 0.04		3.5248	Error Magnification	
**Total Fusion Age**			6.47381	± 0.00235	**18.98**	**± 0.02**		31	1.98 ± 0.01
				± 0.04%		**± 0.13%**			
					Full External Error ± 0.43				
					Analytical Error ± 0.01				
**Normal Isochron**	**169.90**	**± 510.96**	6.73018	± 0.23684	**19.72**	**± 0.69**	16.49	30.18	
Error Chron		**########**		± 3.52%		**± 3.50%**	0%	4	
					Full External Error ± 0.82		3.00	2σ Confidence Limit	
					Analytical Error ± 0.69		4.0609	Error Magnification	
**Inverse Isochron**	**156.54**	**± 203.17**	6.73639	± 0.23672	**19.74**	**± 0.69**	16.46	30.18	
Error Chron		**########**		± 3.51%		**± 3.50%**	0%	4	
					Full External Error ± 0.82		3.00	2σ Confidence Limit	
					Analytical Error ± 0.69		4.0565	Error Magnification	
							0%	Spreading Factor	

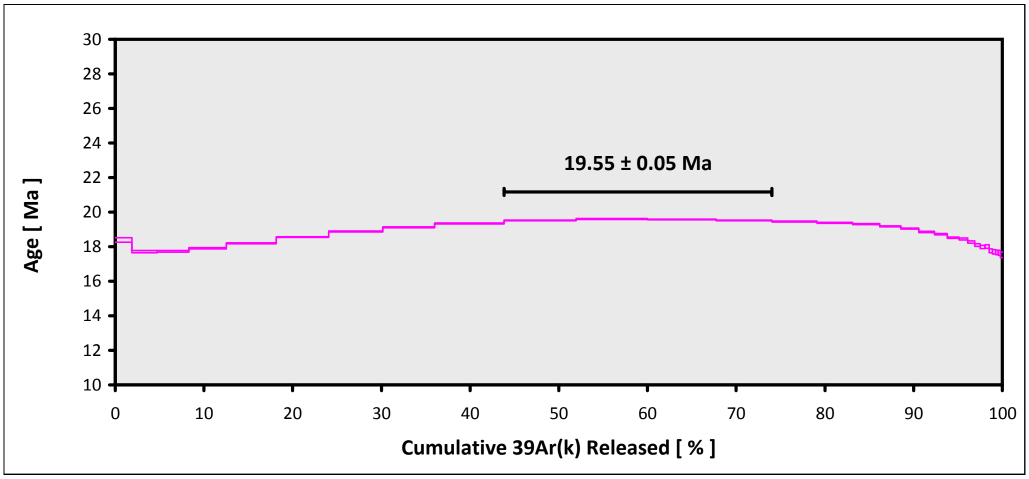

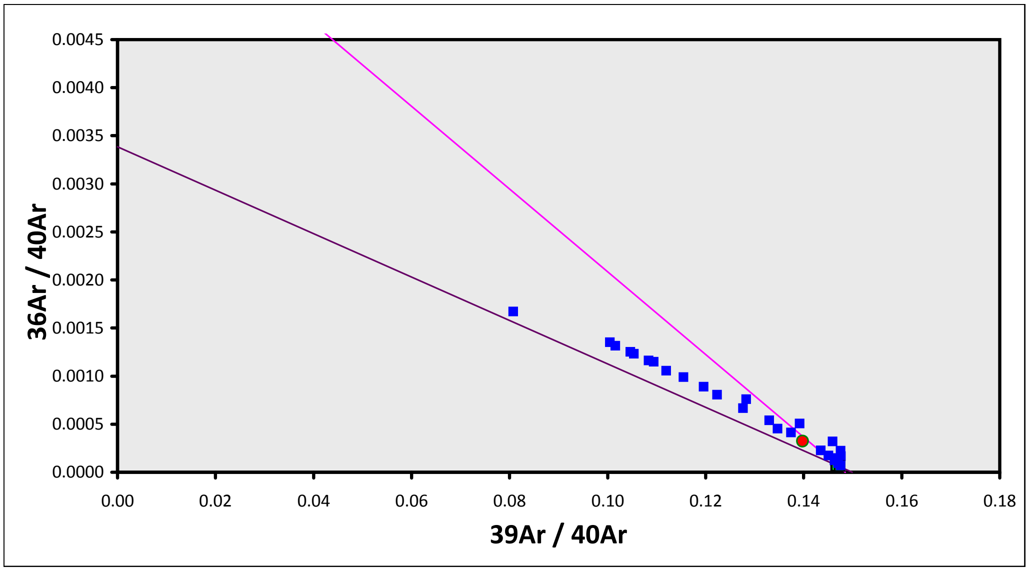

EXP#17D32017 > D16-101 > Plagioclase > IRVING (17-21)
PANAMA > PANAMA CANAL
17-OSU-06 (6F7-17) > Incremental Heating > Dan Miggins

Information on Analysis and Constants Used in Calculations

Project = **IRVING (17-21)**
Sample = **D16-101**
Material = **Plagioclase**
Location = **Panama Canal**
Region = **Panama**
Analyst = **Dan Miggins**
Irradiation = **17-OSU-06 (6F7-17)**
Position = **X: 0 | Y: 0 | Z/H: 9.268932 mm**
FCT-NM Age = **28.201 ± 0.023 Ma**
FCT-NM Reference = **Kuiper et al (2008)**
FCT-NM 40Ar/39Ar Ratio = **9.58814 ± 0.01026**
FCT-NM J-value = **0.00163925 ± 0.00000175**
Air Shot 40Ar/36Ar = **302.9860 ± 0.4272**
Air Shot MDF = **0.99381516 ± 0.00067326 (LIN)**
Experiment Type = **Incremental Heating**
Extraction Method = **Bulk Laser Heating**
Heating = **64 sec**
Isolation = **6.00 min**
Instrument = **ARGUS-VI-D**
Preferred Age = **Plateau Age**
Age Classification = **Crystallization Age**
IGSN = Undefined
Rock Class = Undefined
Lithology = Undefined
Lat-Lon = Undefined - Undefined
Age Equations = **Min et al. (2000)**
Negative Intensities = **Allowed**
Collector Calibrations = **36Ar**
Decay 40K = **5.530 ± 0.048 E-10 1/a**
Decay 39Ar = **2.940 ± 0.016 E-07 1/h**
Decay 37Ar = **8.230 ± 0.012 E-04 1/h**
Decay 36Cl = **2.257 ± 0.015 E-06 1/a**
Decay 40K(EC,β^+) = **0.580 ± 0.009 E-10 1/a**
Decay 40K(β^-) = **4.950 ± 0.043 E-10 1/a**
Atmospheric 40/36(a) = **295.50**
Atmospheric 38/36(a) = **0.1869**
Production 39/37(ca) = **0.0006425 ± 0.0000059**
Production 38/37(ca) = **0.0001800 ± 0.0000173**
Production 36/37(ca) = **0.0002703 ± 0.0000005**
Production 40/39(k) = **0.000607 ± 0.000059**
Production 38/39(k) = **0.012077 ± 0.000011**
Production 36/38(cl) = **262.80 ± 1.71**
Scaling Ratio K/Ca = **0.430**
Abundance Ratio 40K/K = **1.1700 ± 0.0100 E-04**
Atomic Weight K = **39.0983 ± 0.0001 g**

Nice Plateau

Results

Results	40(a)/36(a) ± 2σ		40(r)/39(k) ± 2σ		Age ± 2σ (Ma)		MSWD	39Ar(k) (%,n)	K/Ca ± 2σ
**Age Plateau**			7.14429	± 0.01555<br>± 0.22%	**21.05**	**± 0.06**<br>**± 0.30%**	1.33<br>13%	100.00<br>24	0.0326 ± 0.0005
				Full External Error		± 0.48	1.59	2σ Confidence Limit	
				Analytical Error		± 0.05	1.1541	Error Magnification	
**Total Fusion Age**			7.12703	± 0.03167<br>± 0.44%	**21.00**	**± 0.10**<br>**± 0.49%**		24	0.0327 ± 0.0001
				Full External Error		± 0.48			
				Analytical Error		± 0.09			
**Normal Isochron**	**295.14**	**± 0.84**<br>**± 0.28%**	7.14682	± 0.01762<br>± 0.25%	**21.06**	**± 0.07**<br>**± 0.32%**	1.34<br>13%	100.00<br>24	
				Full External Error		± 0.48	1.60	2σ Confidence Limit	
				Analytical Error		± 0.05	1.1571	Error Magnification	
**Inverse Isochron**	**295.13**	**± 0.84**<br>**± 0.29%**	7.14810	± 0.01769<br>± 0.25%	**21.07**	**± 0.07**<br>**± 0.33%**	1.35<br>13%	100.00<br>24	
				Full External Error		± 0.48	1.60	2σ Confidence Limit	
				Analytical Error		± 0.05	1.1604	Error Magnification	
							83%	Spreading Factor	

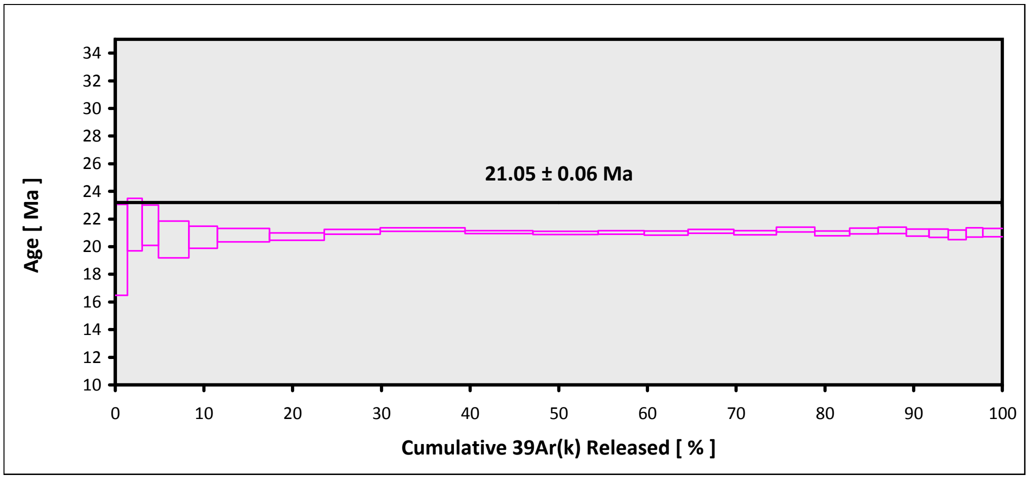

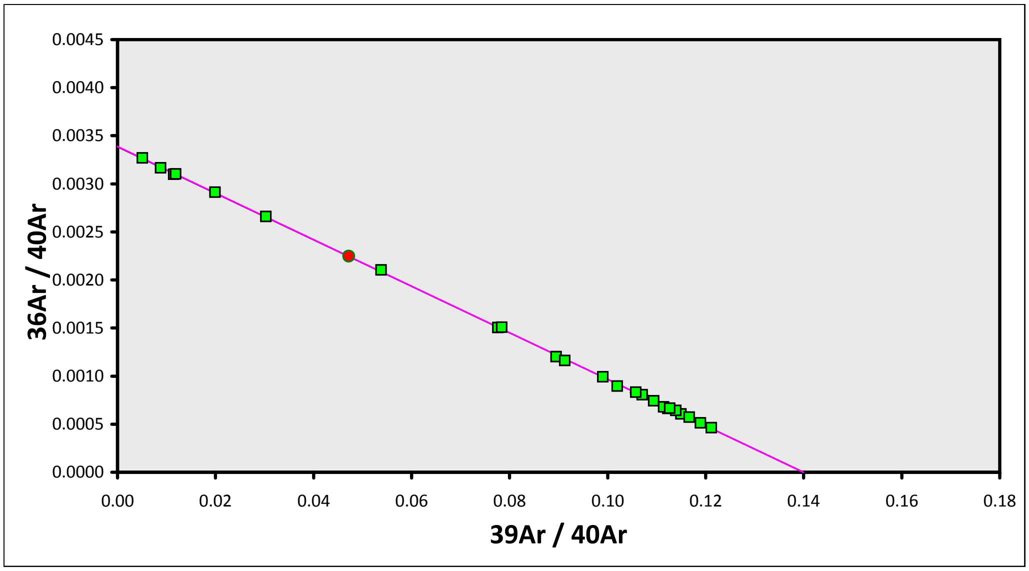

EXP#17D32732 > D16-101 > Groundmass > IRVING (17-21) PANAMA > PANAMA CANAL 17-OSU-06 (6X23-17) > Incremental Heating > Dan Miggins

Information on Analysis and Constants Used in Calculations

Project = **IRVING (17-21)**
Sample = **D16-101**
Material = **Groundmass**
Location = **Panama Canal**
Region = **Panama**
Analyst = **Dan Miggins**
Irradiation = **17-OSU-06 (6X23-17)**
Position = **X: 0 | Y: 0 | Z/H: 27.62394 mm**
FCT-NM Age = **28.201 ± 0.023 Ma**
FCT-NM Reference = **Kuiper et al (2008)**
FCT-NM 40Ar/39Ar Ratio = **9.67869 ± 0.00610**
FCT-NM J-value = **0.00162392 ± 0.00000102**
Air Shot 40Ar/36Ar = **302.5190 ± 0.4024**
Air Shot MDF = **0.99419204 ± 0.00066451 (LIN)**
Experiment Type = **Incremental Heating**
Extraction Method = **Bulk Laser Heating**
Heating = **64 sec**
Isolation = **6.00 min**
Instrument = **ARGUS-VI-D**
Preferred Age = **Mini Plateau**
Age Classification = **Crystallization Age**
IGSN = Undefined
Rock Class = Undefined
Lithology = Undefined
Lat-Lon = Undefined - Undefined
Age Equations = **Min et al. (2000)**
Negative Intensities = **Allowed**
Collector Calibrations = **36Ar**
Decay 40K = **5.530 ± 0.048 E-10 1/a**
Decay 39Ar = **2.940 ± 0.016 E-07 1/h**
Decay 37Ar = **8.230 ± 0.012 E-04 1/h**
Decay 36Cl = **2.257 ± 0.015 E-06 1/a**
Decay 40K(EC,β^+) = **0.580 ± 0.009 E-10 1/a**
Decay 40K(β^-) = **4.950 ± 0.043 E-10 1/a**
Atmospheric 40/36(a) = **295.50**
Atmospheric 38/36(a) = **0.1869**
Production 39/37(ca) = **0.0006425 ± 0.0000059**
Production 38/37(ca) = **0.0001800 ± 0.0000173**
Production 36/37(ca) = **0.0002703 ± 0.0000005**
Production 40/39(k) = **0.000607 ± 0.000059**
Production 38/39(k) = **0.012077 ± 0.000011**
Production 36/38(cl) = **262.80 ± 1.71**
Scaling Ratio K/Ca = **0.430**
Abundance Ratio 40K/K = **1.1700 ± 0.0100 E-04**
Atomic Weight K = **39.0983 ± 0.0001 g**

Mini-Plateau- Disturbed Age Spectrum Ar-loss

Results

Results	40(a)/36(a) ± 2σ		40(r)/39(k) ± 2σ		Age ± 2σ (Ma)		MSWD	39Ar(k) (%,n)	K/Ca ± 2σ
**Age Plateau**			7.19231	± 0.00754<br>± 0.10%	**21.00**	**± 0.03<br>± 0.16%**	1.63<br>18%	20.51<br>4	1.77 ± 0.11
					Full External Error	± 0.47	2.63	2σ Confidence Limit	
					Analytical Error	± 0.02	1.2753	Error Magnification	
**Total Fusion Age**			7.02834	± 0.00506<br>± 0.07%	**20.52**	**± 0.03<br>± 0.14%**		31	1.56 ± 0.00
					Full External Error	± 0.46			
					Analytical Error	± 0.01			
**Normal Isochron**	**286.42**	**± 11.36<br>± 3.97%**	7.21873	± 0.03366<br>± 0.47%	**21.07**	**± 0.10<br>± 0.48%**	1.13<br>32%	20.51<br>4	
					Full External Error	± 0.49	3.00	2σ Confidence Limit	
					Analytical Error	± 0.10	1.0619	Error Magnification	
**Inverse Isochron**<br>Clustered Points	**286.76**	**± 11.37<br>± 3.96%**	7.21777	± 0.03367<br>± 0.47%	**21.07**	**± 0.10<br>± 0.48%**	1.13<br>32%	20.51<br>4	
					Full External Error	± 0.48	3.00	2σ Confidence Limit	
					Analytical Error	± 0.10	1.0622	Error Magnification	
							4%	Spreading Factor	

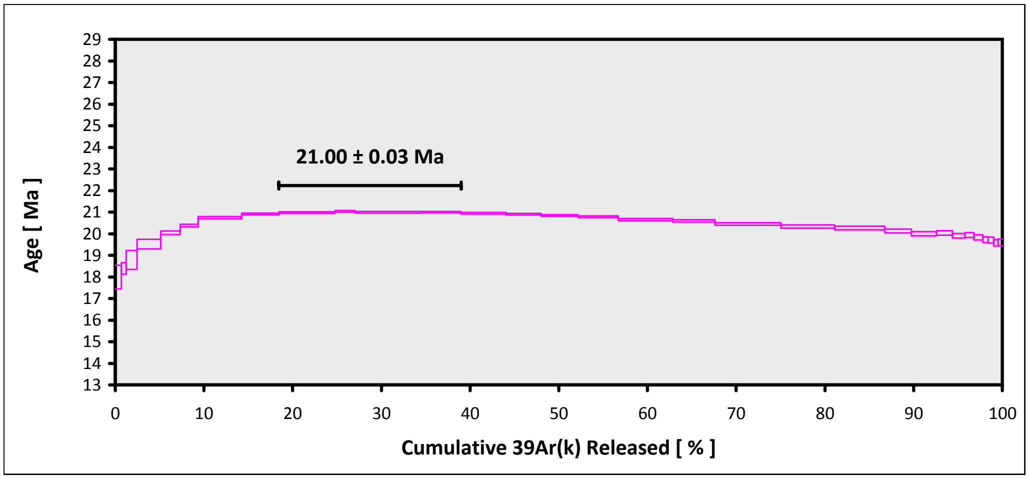

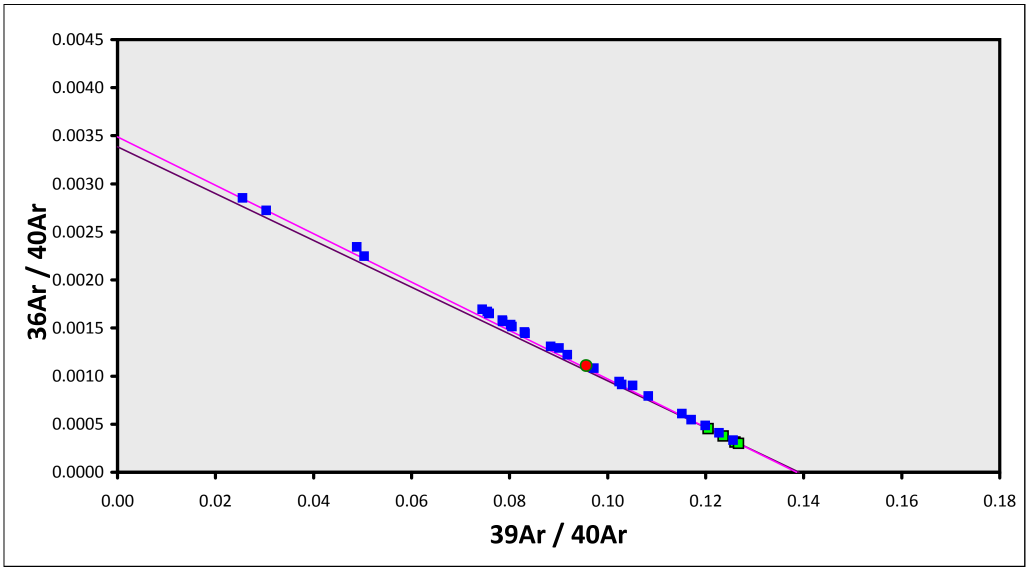

EXP#17D32783 > D16-007 > Groundmass > IRVING (17-21) PANAMA > PANAMA CANAL 17-OSU-06 (6X21-17) > Incremental Heating > Dan Miggins

Information on Analysis and Constants Used in Calculations

Project = **IRVING (17-21)**
Sample = **D16-007**
Material = **Groundmass**
Location = **Panama Canal**
Region = **Panama**
Analyst = **Dan Miggins**
Irradiation = **17-OSU-06 (6X21-17)**
Position = **X: 0 | Y: 0 | Z/H: 25.19495 mm**
FCT-NM Age = **28.201 ± 0.023 Ma**
FCT-NM Reference = **Kuiper et al (2008)**
FCT-NM 40Ar/39Ar Ratio = **9.65768 ± 0.00608**
FCT-NM J-value = **0.00162745 ± 0.00000103**
Air Shot 40Ar/36Ar = **302.7210 ± 0.4026**
Air Shot MDF = **0.99402888 ± 0.00066407 (LIN)**
Experiment Type = **Incremental Heating**
Extraction Method = **Bulk Laser Heating**
Heating = **64 sec**
Isolation = **6.00 min**
Instrument = **ARGUS-VI-D**
Preferred Age = **Mini Plateau**
Age Classification = **Crystallization Age**
IGSN = Undefined
Rock Class = Undefined
Lithology = Undefined
Lat-Lon = Undefined - Undefined
Age Equations = **Min et al. (2000)**
Negative Intensities = **Allowed**
Collector Calibrations = **36Ar**
Decay 40K = **5.530 ± 0.048 E-10 1/a**
Decay 39Ar = **2.940 ± 0.016 E-07 1/h**
Decay 37Ar = **8.230 ± 0.012 E-04 1/h**
Decay 36Cl = **2.257 ± 0.015 E-06 1/a**
Decay 40K(EC,β^+) = **0.580 ± 0.009 E-10 1/a**
Decay 40K(β^-) = **4.950 ± 0.043 E-10 1/a**
Atmospheric 40/36(a) = **295.50 ± 0.70**
Atmospheric 38/36(a) = **0.1869**
Production 39/37(ca) = **0.0006425 ± 0.0000059**
Production 38/37(ca) = **0.0001800 ± 0.0000173**
Production 36/37(ca) = **0.0002703 ± 0.0000005**
Production 40/39(k) = **0.000607 ± 0.000059**
Production 38/39(k) = **0.012077 ± 0.000011**
Production 36/38(cl) = **262.80 ± 1.71**
Scaling Ratio K/Ca = **0.430**
Abundance Ratio 40K/K = **1.1700 ± 0.0100 E-04**
Atomic Weight K = **39.0983 ± 0.0001 g**

Mini-Plateau_ 39Ar recoil

Results

Results	40(a)/36(a) ± 2σ	40(r)/39(k) ± 2σ	Age ± 2σ (Ma)	MSWD	39Ar(k) (%,n)	K/Ca ± 2σ
**Age Plateau**		8.43663 ± 0.03552<br>± 0.42%	**24.66 ± 0.11**<br>**± 0.44%**<br>Full External Error ± 0.57<br>Analytical Error ± 0.10	1.69<br>15%<br>2.41<br>1.2995	31.94<br>5<br>2σ Confidence Limit<br>Error Magnification	0.0266 ± 0.0045
**Total Fusion Age**		8.65982 ± 0.02328<br>± 0.27%	**25.31 ± 0.07**<br>**± 0.29%**<br>Full External Error ± 0.57<br>Analytical Error ± 0.07		31	0.0282 ± 0.0001
**Normal Isochron**	**301.97 ± 11.99**<br>**± 3.97%**	8.35149 ± 0.16243<br>± 1.94%	**24.41 ± 0.47**<br>**± 1.94%**<br>Full External Error ± 0.72<br>Analytical Error ± 0.47	1.80<br>14%<br>2.63<br>1.3424	31.94<br>5<br>2σ Confidence Limit<br>Error Magnification	
**Inverse Isochron**	**302.07 ± 11.96**<br>**± 3.96%**	8.35063 ± 0.16192<br>± 1.94%	**24.41 ± 0.47**<br>**± 1.93%**<br>Full External Error ± 0.72<br>Analytical Error ± 0.47	1.79<br>15%<br>2.63<br>1.3395<br>13%	31.94<br>5<br>2σ Confidence Limit<br>Error Magnification<br>Spreading Factor	

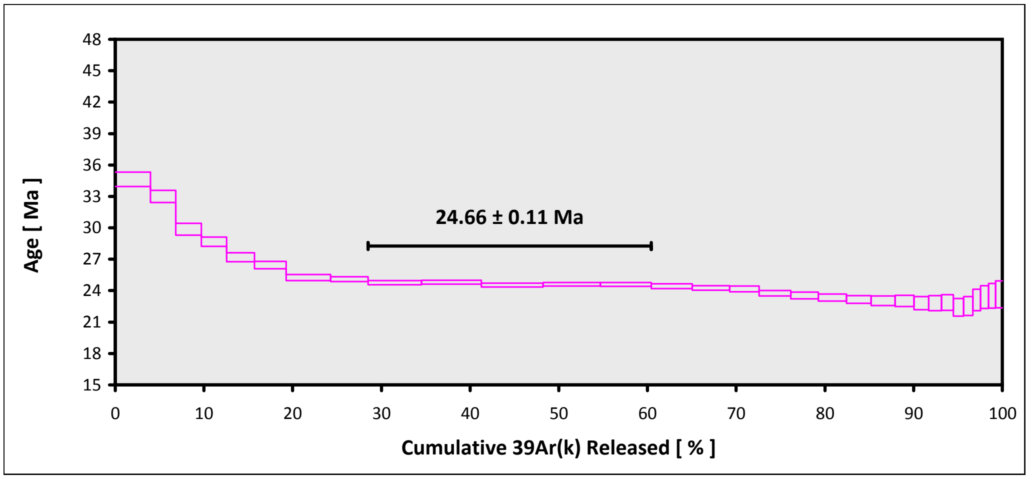

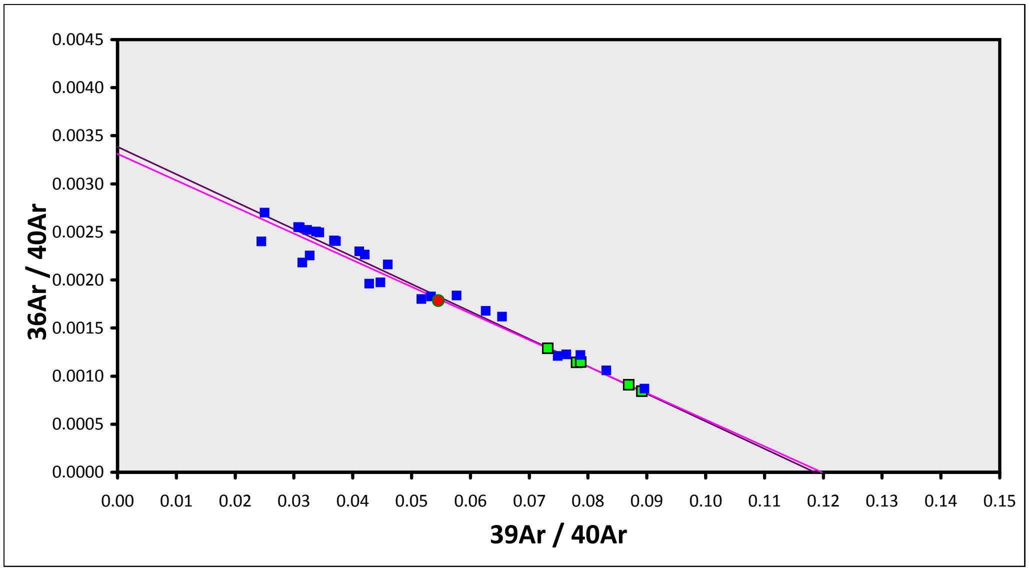

EXP#17D31937 > D16-009 > Plagioclase > IRVING (17-21)
PANAMA > PANAMA CANAL
17-OSU-06 (6F8-17) > Incremental Heating > Dan Miggins

Information on Analysis and Constants Used in Calculations

Project = **IRVING (17-21)**
Sample = **D16-009**
Material = **Plagioclase**
Location = **Panama Canal**
Region = **Panama**
Analyst = **Dan Miggins**
Irradiation = **17-OSU-06 (6F8-17)**
Position = **X: 0 | Y: 0 | Z/H: 10.50825 mm**
FCT-NM Age = **28.201 ± 0.023 Ma**
FCT-NM Reference = **Kuiper et al (2008)**
FCT-NM 40Ar/39Ar Ratio = **9.58918 ± 0.01026**
FCT-NM J-value = **0.00163908 ± 0.00000175**
Air Shot 40Ar/36Ar = **303.2020 ± 0.4427**
Air Shot MDF = **0.99364124 ± 0.00067910 (LIN)**
Experiment Type = **Incremental Heating**
Extraction Method = **Bulk Laser Heating**
Heating = **64 sec**
Isolation = **6.00 min**
Instrument = **ARGUS-VI-D**
Preferred Age = **Plateau Age**
Age Classification = **Crystallization Age**
IGSN = Undefined
Rock Class = Undefined
Lithology = Undefined
Lat-Lon = Undefined - Undefined
Age Equations = **Min et al. (2000)**
Negative Intensities = **Allowed**
Collector Calibrations = **36Ar**
Decay 40K = **5.530 ± 0.048 E-10 1/a**
Decay 39Ar = **2.940 ± 0.016 E-07 1/h**
Decay 37Ar = **8.230 ± 0.012 E-04 1/h**
Decay 36Cl = **2.257 ± 0.015 E-06 1/a**
Decay 40K(EC,β^+) = **0.580 ± 0.009 E-10 1/a**
Decay 40K(β^-) = **4.950 ± 0.043 E-10 1/a**
Atmospheric 40/36(a) = **295.50**
Atmospheric 38/36(a) = **0.1869**
Production 39/37(ca) = **0.0006425 ± 0.0000059**
Production 38/37(ca) = **0.0001800 ± 0.0000173**
Production 36/37(ca) = **0.0002703 ± 0.0000005**
Production 40/39(k) = **0.000607 ± 0.000059**
Production 38/39(k) = **0.012077 ± 0.000011**
Production 36/38(cl) = **262.80 ± 1.71**
Scaling Ratio K/Ca = **0.430**
Abundance Ratio 40K/K = **1.1700 ± 0.0100 E-04**
Atomic Weight K = **39.0983 ± 0.0001 g**

Nice Plateau

Results

Results	40(a)/36(a) ± 2σ	40(r)/39(k) ± 2σ	Age ± 2σ (Ma)	MSWD	39Ar(k) (%,n)	K/Ca ± 2σ
**Age Plateau**		8.70025 ± 0.06259 ± 0.72%	**25.61 ± 0.19 ± 0.75%**	0.82	100.00	0.0068 ± 0.0001
				71%	24	
			Full External Error ± 0.61	1.59	2σ Confidence Limit	
			Analytical Error ± 0.18	1.0000	Error Magnification	
**Total Fusion Age**		8.84395 ± 0.37085 ± 4.19%	**26.03 ± 1.08 ± 4.17%**		24	0.0068 ± 0.0000
			Full External Error ± 1.23			
			Analytical Error ± 1.08			
**Normal Isochron**	**295.92 ± 0.65 ± 0.22%**	8.67398 ± 0.07504 ± 0.87%	**25.53 ± 0.23 ± 0.88%**	0.78	100.00	
				76%	24	
			Full External Error ± 0.62	1.60	2σ Confidence Limit	
			Analytical Error ± 0.22	1.0000	Error Magnification	
**Inverse Isochron**	**295.91 ± 0.65 ± 0.22%**	8.67611 ± 0.07508 ± 0.87%	**25.53 ± 0.23 ± 0.89%**	0.77	100.00	
				77%	24	
			Full External Error ± 0.62	1.60	2σ Confidence Limit	
			Analytical Error ± 0.22	1.0000	Error Magnification	
				49%	Spreading Factor	

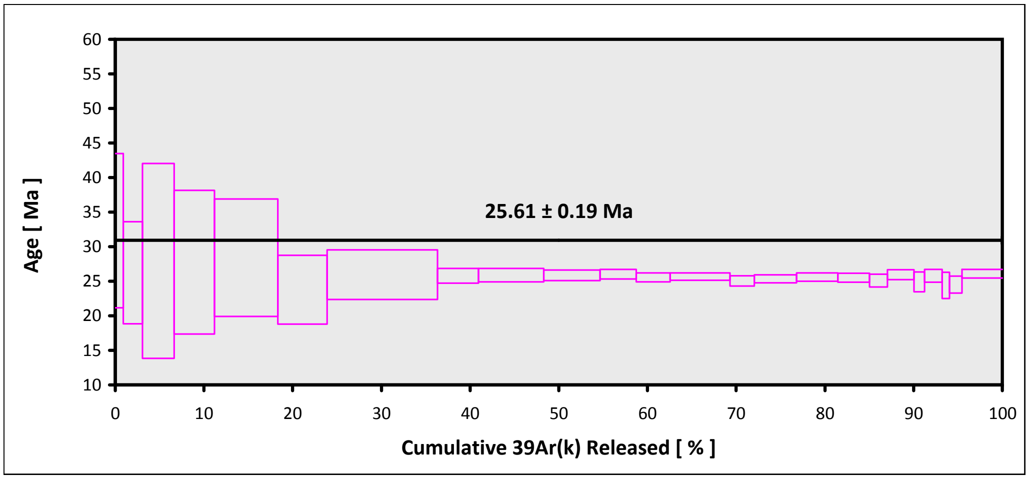

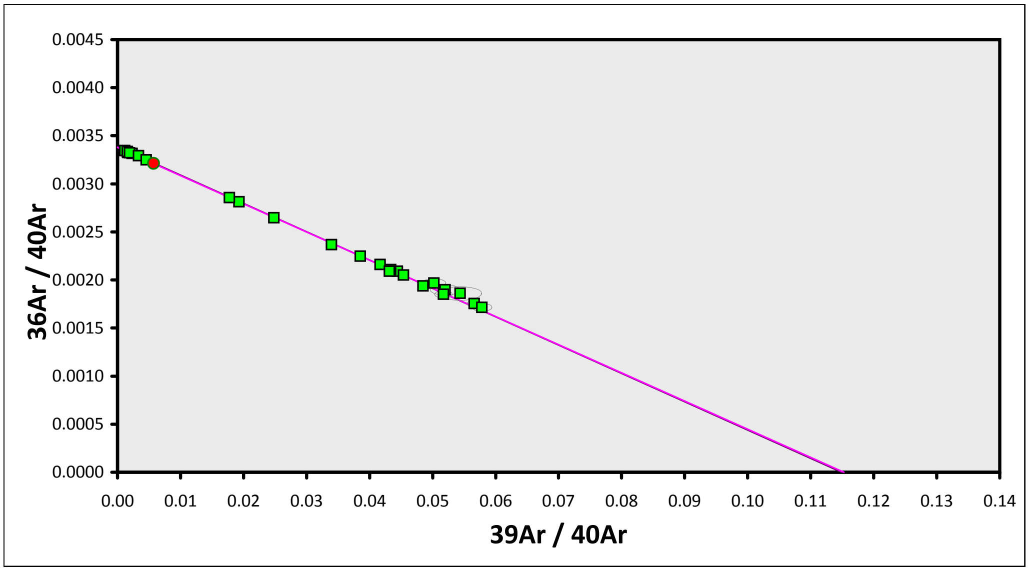

EXP#17D32627 > D16-009 > Groundmass > IRVING (17-21)
PANAMA > PANAMA CANAL
17-OSU-06 (6X8-17) > Incremental Heating > Dan Miggins

Information on Analysis and Constants Used in Calculations

Project = **IRVING (17-21)**
Sample = **D16-009**
Material = **Groundmass**
Location = **Panama Canal**
Region = **Panama**
Analyst = **Dan Miggins**
Irradiation = **17-OSU-06 (6X8-17)**
Position = **X: 0 | Y: 0 | Z/H: 10.36062 mm**
FCT-NM Age = **28.201 ± 0.023 Ma**
FCT-NM Reference = **Kuiper et al (2008)**
FCT-NM 40Ar/39Ar Ratio = **9.58426 ± 0.00613**
FCT-NM J-value = **0.00163992 ± 0.00000105**
Air Shot 40Ar/36Ar = **302.9250 ± 0.1666**
Air Shot MDF = **0.99386433 ± 0.00059410 (LIN)**
Experiment Type = **Incremental Heating**
Extraction Method = **Bulk Laser Heating**
Heating = **64 sec**
Isolation = **6.00 min**
Instrument = **ARGUS-VI-D**
Preferred Age = **Total Fusion**
Age Classification = **Crystallization Age**
IGSN = Undefined
Rock Class = Undefined
Lithology = Undefined
Lat-Lon = Undefined - Undefined
Age Equations = **Min et al. (2000)**
Negative Intensities = **Allowed**
Collector Calibrations = **36Ar**
Decay 40K = **5.530 ± 0.048 E-10 1/a**
Decay 39Ar = **2.940 ± 0.016 E-07 1/h**
Decay 37Ar = **8.230 ± 0.012 E-04 1/h**
Decay 36Cl = **2.257 ± 0.015 E-06 1/a**
Decay 40K(EC,β^+) = **0.580 ± 0.009 E-10 1/a**
Decay 40K(β^-) = **4.950 ± 0.043 E-10 1/a**
Atmospheric 40/36(a) = **295.50**
Atmospheric 38/36(a) = **0.1869**
Production 39/37(ca) = **0.0006425 ± 0.0000059**
Production 38/37(ca) = **0.0001800 ± 0.0000173**
Production 36/37(ca) = **0.0002703 ± 0.0000005**
Production 40/39(k) = **0.000607 ± 0.000059**
Production 38/39(k) = **0.012077 ± 0.000011**
Production 36/38(cl) = **262.80 ± 1.71**
Scaling Ratio K/Ca = **0.430**
Abundance Ratio 40K/K = **1.1700 ± 0.0100 E-04**
Atomic Weight K = **39.0983 ± 0.0001 g**

Total Fusion Age only- significant 39Ar recoil

Results

Results	40(a)/36(a) ± 2σ	40(r)/39(k) ± 2σ	Age ± 2σ (Ma)	MSWD	39Ar(k) (%,n)	K/Ca ± 2σ
**Age Plateau**<br>Cannot Calculate						
**Total Fusion Age**		8.85817 ± 0.00466<br>± 0.05%	**26.08 ± 0.04**<br>**± 0.14%**<br>Full External Error ± 0.59<br>Analytical Error ± 0.01		31	0.117 ± 0.000
**Normal Isochron**<br>Cannot Calculate						
**Inverse Isochron**<br>Cannot Calculate						

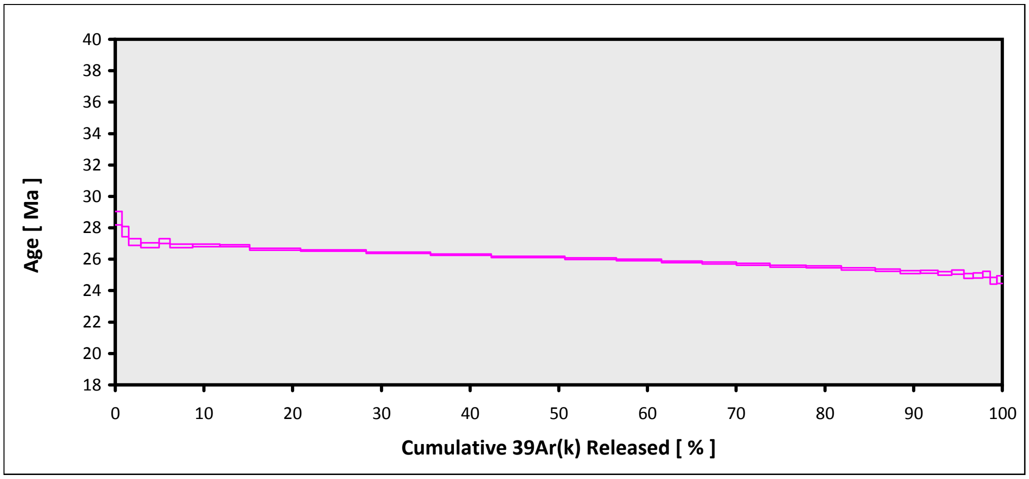

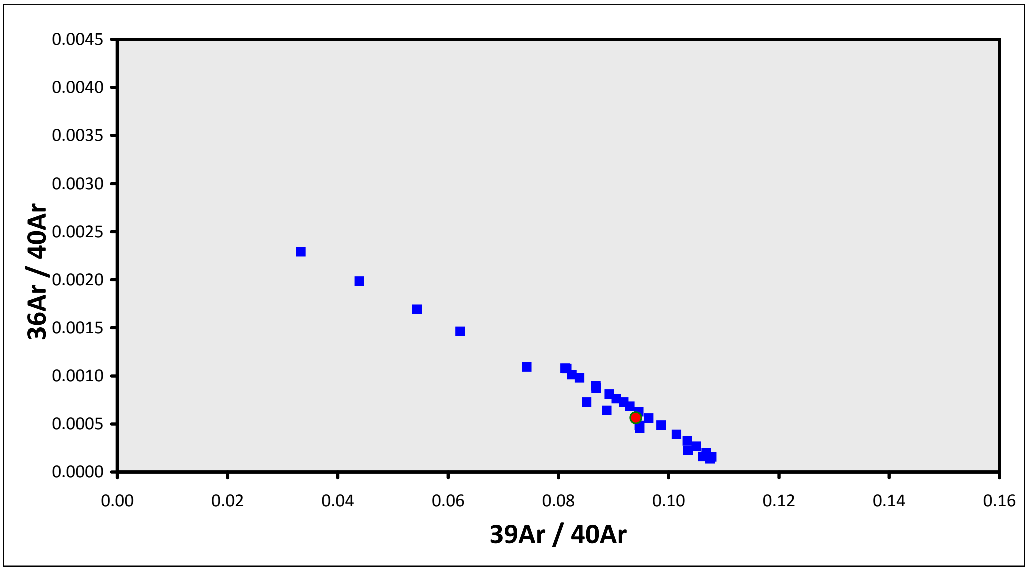

Supplement: Supplementary file 4 — Supplementary Dataset 4 [file 41598_2018_37790_MOESM4_ESM.pdf]
